# Natural Image Reconstruction From fMRI Using Deep Learning: A Survey

**DOI:** 10.3389/fnins.2021.795488

**Published:** 2021-12-20

**Authors:** Zarina Rakhimberdina, Quentin Jodelet, Xin Liu, Tsuyoshi Murata

**Affiliations:** ^1^Department of Computer Science, Tokyo Institute of Technology, Tokyo, Japan; ^2^AIST-Tokyo Tech Real World Big-Data Computation Open Innovation Laboratory, Tokyo, Japan; ^3^Artificial Intelligence Research Center, National Institute of Advanced Industrial Science and Technology, Tokyo, Japan; ^4^Digital Architecture Research Center, National Institute of Advanced Industrial Science and Technology, Tokyo, Japan

**Keywords:** natural image reconstruction, fMRI, brain decoding, neural decoding, deep learning

## Abstract

With the advent of brain imaging techniques and machine learning tools, much effort has been devoted to building computational models to capture the encoding of visual information in the human brain. One of the most challenging brain decoding tasks is the accurate reconstruction of the perceived natural images from brain activities measured by functional magnetic resonance imaging (fMRI). In this work, we survey the most recent deep learning methods for natural image reconstruction from fMRI. We examine these methods in terms of architectural design, benchmark datasets, and evaluation metrics and present a fair performance evaluation across standardized evaluation metrics. Finally, we discuss the strengths and limitations of existing studies and present potential future directions.

## 1. Introduction

### 1.1. Visual Decoding Using fMRI

Many brain imaging studies focus on decoding how the human brain represents information about the outer world. Considering that the majority of external sensory information is processed by the human visual system (Logothetis and Sheinberg, 1996), a need for deeper understanding of visual information processing in the human brain encourages building complex computational models that can characterize the content of visual stimuli. This problem is referred to as *human visual decoding* of perceived images and has gained increasing attention.

A great advancement in recent neuroscience research has been achieved through functional magnetic resonance imaging (fMRI) (Poldrack and Farah, 2015; Nestor et al., 2020). The fMRI technique captures neural activity in the brain by measuring variations in blood oxygen levels (Ogawa et al., 1990; Bandettini, 2012). Among the various brain imaging techniques, fMRI is non-invasive and has a high spatial resolution. These characteristics allow fMRI to be used in a wide range of problems, including neurological disorder diagnosis (Rakhimberdina et al., 2020; Zhang et al., 2020) and human visual decoding (Haxby et al., 2001; Kamitani and Tong, 2005; Horikawa and Kamitani, 2017). The recent progress in human visual decoding has shown that beyond merely encoding the information about visual stimuli (Poldrack and Farah, 2015), brain activity captured by fMRI can be used to reconstruct visual stimuli information (Kay et al., 2008; Roelfsema et al., 2018).

**Figure 1:**
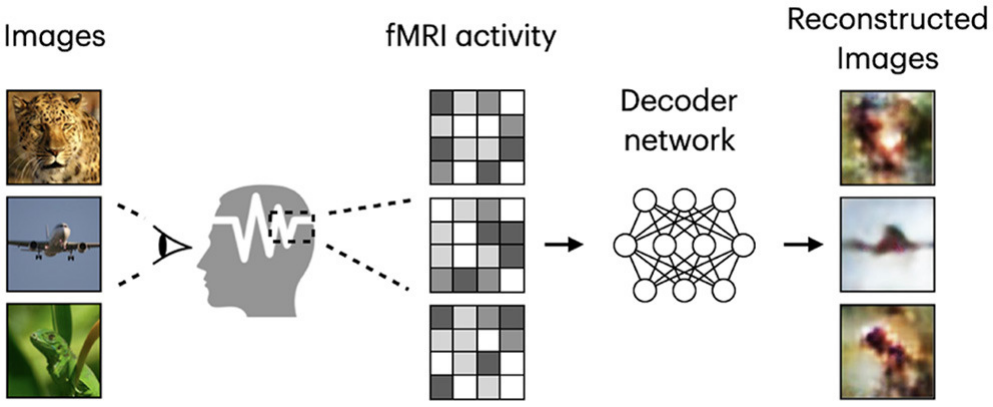
Framework diagram for natural image reconstruction task. Images are from ImageNet dataset (Deng et al., 2009).

Based on the target task, human visual decoding can be categorized into stimuli category classification, stimuli identification, and reconstruction (Naselaris et al., 2011). In classification, brain activity is used to predict discrete object categories of the presented stimuli (Haxby et al., 2001; Horikawa and Kamitani, 2017). The goal of identification is to identify a specific stimulus corresponding to the given pattern of brain activity from a known set of stimuli images (Kay et al., 2008; Naselaris et al., 2011). In both identification and reconstruction, we aim to recover image-specific details, such as object position, size, and angle. However, reconstruction is a more challenging task, in which a replica of the stimulus image needs to be generated for a given fMRI signal (see Figure 1). Furthermore, stimulus-related information encoded in the fMRI activity, which allows high-accuracy identification, may only partially characterize stimuli images and thus be insufficient for proper image reconstruction (Kay et al., 2008; St-Yves and Naselaris, 2018). With the development of sophisticated image reconstruction methods and the increasing amount of brain imaging data, more attention has been directed toward visual stimuli reconstruction from fMRI activity in the visual cortex (Miyawaki et al., 2008; Naselaris et al., 2009; van Gerven et al., 2010). fMRI-based visual reconstruction can improve our understanding of the brain's visual processing mechanisms, and researchers can incorporate this knowledge into the development of brain–computer interfaces.

### 1.2. Natural Image Reconstruction

The variety of visual stimuli used in visual reconstruction tasks can range from simple low-level detail images, such as Gabor wavelets and domino patterns (Thirion et al., 2006), to more elaborate images depicting alphabetical characters, digits (van Gerven et al., 2010; Schoenmakers et al., 2013), natural objects, and scenes (Haxby et al., 2001; Horikawa and Kamitani, 2017). The image reconstruction task for low-level detail stimuli does not require expressive models, and linear mapping is usually sufficient for learning effective reconstruction (Miyawaki et al., 2008). Among the variety of visual stimuli, natural images are considered the most challenging, as they require accurate reconstruction of color, shape, and higher-level perceptual features.

Similar to Shen et al. (2019b), we refer to the task of visual stimuli reconstruction from fMRI as *natural image reconstruction*, where stimuli are drawn from a database of natural images. The goal of neural decoding models is to learn a mapping function f:V→X, where X and V denote two sets corresponding to stimulus images and fMRI activity patterns extracted from the visual cortex. A framework diagram for visual reconstruction is shown in Figure 1.

The main challenges of natural image reconstruction include the following. First, the reconstruction quality must be good enough to capture the similarity between reconstructed and original images on multiple levels. In contrast to low-resolution image stimuli, such as shape or character patterns, good-quality reconstruction of natural images requires that both lower-level details and high-level semantic information be preserved. Second, brain's visual representations are invariant to different objects or image details, which is essential for object recognition, but imply that brain activation patterns are not necessarily unique for a given stimulus object (Quiroga et al., 2005; St-Yves and Naselaris, 2018). Finally, the lack of a standardized evaluation procedure for assessing the reconstruction quality makes it difficult to compare the existing methods. In this work, we will primarily focus on the solution to the third challenge.

#### 1.2.1. Contributions

The topic of natural image reconstruction from fMRI is relatively new and has attracted much interest over the last few years. The related surveys on the field of natural encoding and decoding of visual input give a broad overview of the existing techniques to extract information from the brain (Roelfsema et al., 2018; Nestor et al., 2020) and focus on the traditional machine learning methods (Chen et al., 2014). To our knowledge, there is no comprehensive survey on the topic of natural image reconstruction from fMRI using deep learning. Given the lack of a standardized evaluation process in terms of the benchmark dataset and standard metrics, our main contribution is to provide the research community with a fair performance comparison for existing methods.

In this survey, we provide an overview of the deep learning-based natural image reconstruction methods. We discuss the differences in architecture, learning paradigms, and advantages of deep learning models over traditional methods. In addition, we review the evaluation metrics and compare models on the same benchmark: the same metrics and the same dataset parameters. The proposed standardized evaluation on a common set of metrics offers an opportunity to fairly evaluate and track new emerging methods in the field.

The rest of this paper is organized as follows. In sections 2 and 3, we introduce popular publicly available datasets for natural image reconstruction and review recent state-of-the-art deep learning models for natural image reconstruction, respectively. Then, we provide an overview of the evaluation metrics in section 4, and presents a fair comparative evaluation of the methods in section 5. Finally, we discuss the main challenges and possible future directions of this work in section 6. Section 7 concludes the paper.

## 2. Benchmark Datasets

This section summarizes the publicly available benchmark datasets used in deep learning-based natural image reconstruction from fMRI activity. While there exist a variety of datasets used for stimuli reconstruction, such as binary contrast patterns (BCP) (Miyawaki et al., 2008), 69 dataset of handwritten digits (van Gerven et al., 2010), BRAINS dataset of handwritten characters (Schoenmakers et al., 2013), we focus on the datasets with higher level of perceptual complexity of presented stimuli: dataset of faces, grayscale natural images, and natural images from Imagenet. Each sample of these datasets represents a labeled pair—fMRI recording paired with the relevant stimuli image. Several distinctive characteristics of each dataset are presented in Table 1.

**Table 1:** Characteristics of benchmark datasets.

**References**	**Dataset**		**Number of subjects**	**Image stimuli train/test**	**Repetition time train/test**	**ROIs**
VanRullen and Reddy (2019)	*Faces*		4	88/20	n/a	n/a
Kay et al. (2008)	*vim-1*		2	1,750/120	2/13	V1, V2, V3, V4, LO
Horikawa and Kamitani (2017)	*Generic object decoding*		5	1,200/50	1/35	V1, V2, V3, V4, LOC, FFA, PPA
Shen et al. (2019b)	*Deep image reconstruction*	Natural images	3	1,200/50	5/24	
		Artificial shapes	3	0/40	0/20	
		Alphabetical letters	3	0/10	0/12	

**Figure 2:**
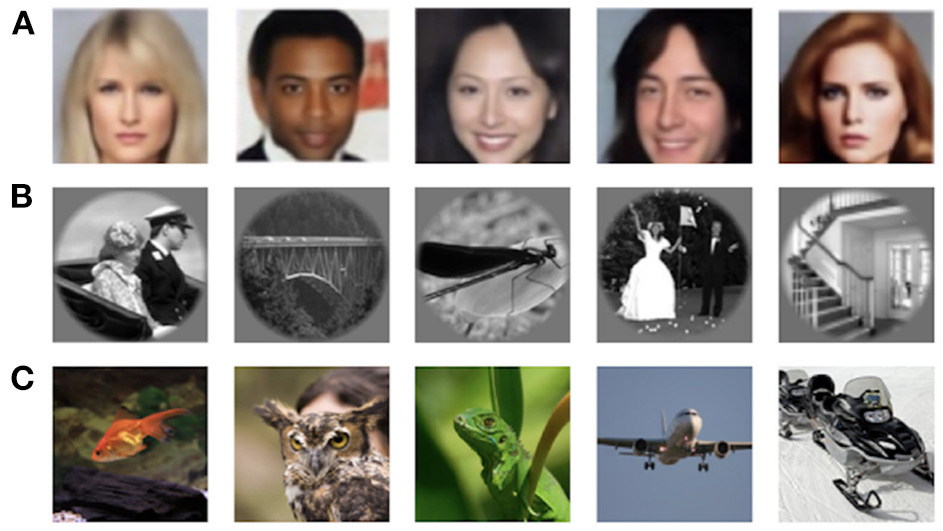
Samples for natural stimuli: **(A)** images from Faces dataset (VanRullen and Reddy, 2019); **(B)** grayscale natural images from vim-1 dataset (Kay et al., 2008); **(C)** natural images from GOD (Horikawa and Kamitani, 2017) and DIR (Shen et al., 2019b) datasets.

**Faces**. VanRullen and Reddy (2019) used facial stimuli to reconstruct human faces from fMRI activity using deep neural networks^1^. The facial stimuli were drawn randomly from the CelebA dataset (Liu et al., 2015), and four healthy subjects participated in the experiment. The samples of stimuli images are shown in Figure 2A.

**vim-1 dataset of grayscale natural images** was acquired to study how natural images are represented by the human visual system^2^ (Kay et al., 2008). The stimuli comprise a set of 1870 grayscale 500 × 500 pixels natural images of real-world objects, animals, and indoor and outdoor scenes (the samples are shown in Figure 2B). Natural images were obtained from the Corel Stock Photo Libraries (Corel Corporation, 1994), the Berkeley database of human segmented natural images^3^ (Martin et al., 2001), and an image collection from the authors. Two healthy subjects with normal or corrected-to-normal vision were involved in the fMRI data acquisition.

**Natural images from imagenet**. Two natural image datasets released by Kamitani Lab are widely used in image reconstruction. The first dataset, also known as the Generic Object Decoding^4^ dataset or GOD for short, was originally used by Horikawa and Kamitani (2017) for the image classification task from the fMRI data and was later adopted for image reconstruction (Beliy et al., 2019; Ren et al., 2021). The dataset consists of pairs of high-resolution 500 × 500 pixels stimuli images (see Figure 2C) and the corresponding fMRI recordings. fMRI scans were obtained from five healthy subjects; the stimuli images were selected from the ImageNet dataset (Deng et al., 2009) and span across 200 object categories.

The second dataset based on the natural image dataset was acquired for the image reconstruction task (Shen et al., 2019a,b). It is publicly available at OpenNeuro^5^, where it is cited as Deep Image Reconstruction. We refer to this dataset as Deep Image Reconstruction or DIR for short. The DIR dataset contains 1,250 stimuli images that are identical to the ones used in GOD. Because of different image presentation experiments, in which training and test image stimuli were repeated 5 and 24 times respectively, the training set of the DIR dataset consists of a larger number of stimuli-fMRI pairs (5 × 1,200 samples) compared to the GOD. Three healthy subjects were involved in the image presentation. An appealing feature of this dataset is that, in addition to natural images, the dataset contains artificial shapes and alphabetical letters. The artificial shapes dataset consists of 40 images—a combination of eight colors and five geometric shapes. The alphabetical letters dataset consists of 10 letters (A, C, E, I, N, O, R, S, T, U) of consistent brightness and color.

## 3. Deep Learning-Based Approaches for Natural Image Reconstruction

Before deep learning, the traditional methods in natural image reconstruction estimated a linear mapping from fMRI signals to hand-crafted image features using linear regression models (Kay et al., 2008; Naselaris et al., 2009; Fujiwara et al., 2013). These methods primarily focus on extracting predefined low-level features from stimulus images, such as local image structures or features of Gabor filters (Beliy et al., 2019; Fang et al., 2020).

In recent years, deep neural networks (DNNs) have significantly advanced computer vision research, replacing models based on hand-crafted features. In particular, DNN models have achieved better accuracy and improved image quality in various computer vision tasks, including image classification (Krizhevsky et al., 2012), image segmentation (Chen et al., 2015), and image restoration (Zhang et al., 2017). In visual decoding tasks using brain imaging data, deep learning approaches have been applied to image classification (Haxby et al., 2001; Horikawa and Kamitani, 2017), object segmentation (Kamnitsas et al., 2017), and natural image reconstruction (Shen et al., 2019a,b). They were shown to be more powerful than traditional methods (Kriegeskorte, 2015; Zhang et al., 2020) primarily due to the multilayer architecture allowing to learn non-linear mappings from brain signals to stimulus images (Beliy et al., 2019; Shen et al., 2019a).

Motivated by the success of deep learning in image generation, many recent studies have widely used DNN models in natural image reconstruction for several reasons. First, the deep learning framework conforms to some degree to the visual encoding–decoding process occurring in the hierarchical regions of the human visual system (Pinto et al., 2009; Krizhevsky et al., 2012; Schrimpf et al., 2018). Second, the application of deep generative models allows the synthesis of high-quality natural-looking images, which is achieved by learning the underlying data distribution (Goodfellow et al., 2014). Additionally, the training process can be aided by models pretrained on larger image datasets (Shen et al., 2019a,b).

In this section, we present the evolution of the state-of-the-art deep learning-based methods for natural image reconstruction. We analyze them in terms of DNN architecture, use of pretraining, and the choice of the dataset. The most popular deep learning models used in natural image reconstruction tasks include non-generative methods such as convolutional neural networks, encoder–decoder-based frameworks (Kingma and Welling, 2014); and generative methods, such as adversarial networks (Goodfellow et al., 2014) and variational autoencoders (Larsen et al., 2016). A comparison of the surveyed methods is presented in Table 2.

**Table 2:** Comparative table of the surveyed works.

**Method**	**References**		**Datasets**	**Loss**	**E2E**	**Pre-training**	**Public code**
SeeligerDCGAN	Seeliger et al., 2018		BRAINS vim-1 GOD	MAE MSE	No	Generator pre-trained on ImageNet (Chrabaszcz et al., 2017), Microsoft COCO Lin et al., 2014, datasets from Maaten (2009) and Schomaker et al. (2000). AlexNet-based Comparator trained on ImageNet.	No
StYvesEBGAN	St-Yves and Naselaris, 2018		vim-1	MSE Adv	No	The denoiser and generator were pretrained on 32 × 32 color images from the CIFAR-10 dataset (Krizhevsky, 2009)	yes
ShenDNN(+DGN)	Shen et al., 2019a		DIR: Natural images, Artificial shapes, Alphabetical letters	MSE	No	VGG-19 pre-trained on ImageNet. Pre-trained DGN (Dosovitskiy and Brox, 2016).	yes
ShenGAN	Shen et al., 2019b		DIR: Natural images, Artificial shapes, Alphabetical letters	MSE Adv	Yes	Caffenet-based Comparator pre-trained on ImageNet	yes
BeliyEncDec	Beliy et al., 2019		GOD vim-1	MSE Cos MAE	No	Pretrained AlexNet-based encoder	yes
VanRullenVAE-GAN	VanRullen and Reddy, 2019		Faces	MSE Adv	No	Pre-trained on CelebA dataset	yes
GazivEncDec	Gaziv et al., 2020		GOD vim-1	MSE Cos MAE	No	Pretrained AlexNet-based encoder	No
QiaoGAN-BVRM	Qiao et al., 2020		vim-1	MSE	No	Generator of BigGAN pre-trained on ImageNet	partially
FangSSGAN	Fang et al., 2020		DIR: Natural images	MAE Adv	No	-	partially
MozafariBigBiGAN	Mozafari et al., 2020		GOD	Adv	No	BigBiGAN pre-trained on ImageNet	No
RenD-VAE/GAN	Ren et al., 2021		BCP 6-9 BRAINS GOD	KL Adv	No	Model pre-trained on external data from ImageNet	No

*E2E represents end-to-end training. Loss denotes the loss function (MAE, mean absolute error; MSE, mean squared error; KL, KL divergence; Adv, adversarial loss; Cos, cosine similarity. The links to the source code are valid as of November, 2021*.

### 3.1. Non-generative Methods

#### 3.1.1. Convolutional Neural Network (CNN)

Compared to a simpler multilayer feed-forward neural network, which disregards the structural information of input images, the CNN has a better feature extraction capability because of the information filtering performed by convolutional layers within a neighborhood of pixels (LeCun et al., 1989). Stacking convolutional layers on top of each other allows learning hierarchical visual features of input images, known as feature abstraction. The lower CNN layers learn low-level details, whereas the higher CNN layers extract global high-level visual information from images (Mahendran and Vedaldi, 2015). The use of CNNs is ubiquitous in image processing tasks, including image reconstruction. Specifically, encoder–decoder (Beliy et al., 2019; Gaziv et al., 2020), U-Net (Fang et al., 2020), generative adversarial network (Goodfellow et al., 2014), and variational autoencoder (Kingma and Welling, 2014) are popular architectures that adopt stacked convolutional layers to extract features at multiple levels.

**Figure 3:**
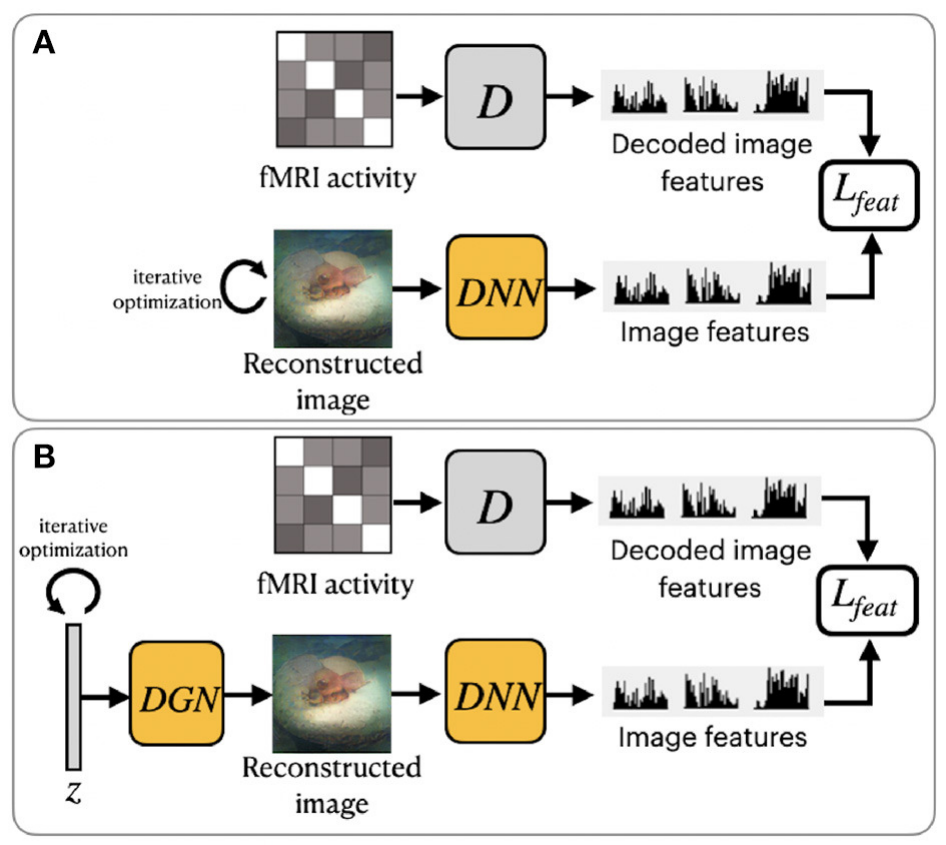
Overview of two variations of frameworks proposed by Shen et al. (2019b): **(A)** ShenDNN and **(B)** ShenDNN+DGN. The yellow color denotes the use of pretrained components.

Shen et al. (2019b) utilized a pretrained VGG-19-based DNN to extract hierarchical features from stimuli images (see Figure 3A). The DNN consists of sixteen convolutional layers followed by three fully connected layers. This method was motivated by the finding that hierarchical image representations obtained from different layers of deep neural network correlate with brain activity in the visual cortex (Eickenberg et al., 2017; Horikawa and Kamitani, 2017). Using this fact, one can establish a hierarchical mapping from fMRI signals in the low/high-level areas of visual cortices to the corresponding low/high-level features from the DNN. For this task, the authors implemented a feature decoder *D* that maps fMRI activity patterns to multilayer DNN features. The decoder *D* is trained on the train set before the reconstruction task, using the method from Horikawa and Kamitani (2017). These decoded fMRI features correspond to the hierarchical image features obtained from DNN. The optimization is performed on the feature space by minimizing the difference between the hierarchical DNN features of the image and multilayer features decoded from fMRI activity.

#### 3.1.2. Deterministic Encoder–Decoder Models

In deep learning, encoder–decoder models are widely used in image-to-image translation (Isola et al., 2017) and sequence-to-sequence models (Cho et al., 2014). They learn the mapping from an input domain to an output domain via a two-stage architecture: an encoder *E* that compresses the input vector **x** to the latent representation **z** = *E*(**x**) and a decoder **y** = *D*(**z**) that produces the output vector **y** from the latent representation **z** (Minaee et al., 2021). The compressed latent representation vector **z** serves as a bottleneck, which encodes a low-dimensional representation of the input. The model is trained to minimize the reconstruction error, which is the difference between the reconstructed output and ground-truth input.

**Figure 4:**
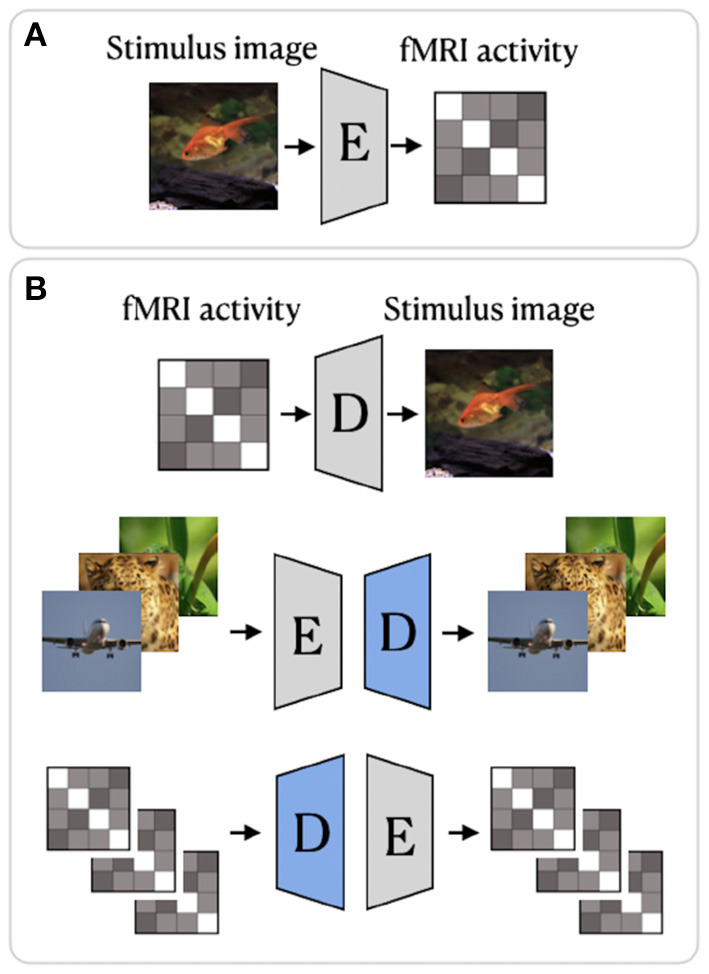
BeliyEncDec framework proposed by Beliy et al. (2019): **(A)** supervised training of the Encoder; **(B)** supervised and self-supervised training of the Decoder. The weights of the Encoder are fixed. The blue color denotes the components of the model trained on external unlabeled data. The image is adapted from Beliy et al. (2019). Images are from ImageNet dataset (Deng et al., 2009).

Beliy et al. (2019) presented a CNN-based encoder–decoder model, where the encoder *E* learns the mapping from stimulus images to the corresponding fMRI activity, and a decoder *D* learns the mapping from fMRI activity to their corresponding images. The framework of this method, which we refer to as BeliyEncDec, is presented in Figure 4. By stacking the encoder and decoder back-to-back, the authors introduced two combined networks *E*-*D* and *D*-*E*, whose inputs and outputs are natural images and fMRI recordings, respectively. This allowed the training to be self-supervised on a larger dataset of unlabeled data. Specifically, 50,000 additional images from the ImageNet validation set and test fMRI recordings without stimulus pairs were used as unlabeled natural images and unlabeled fMRI samples. The authors demonstrated the advantage of their method by achieving competitive results on two natural image reconstruction datasets: Generic Object Decoding (Horikawa and Kamitani, 2017) and vim-1 (Kay et al., 2008). The training was conducted in two steps. In the first step, the encoder *E* builds a mapping from stimulus images to fMRI activity. It utilizes the weights of the first convolutional layer of the pretrained AlexNet (Krizhevsky et al., 2012) and is trained in a supervised manner to predict fMRI activity for input images. In the second step, the trained encoder *E* is fixed, and the decoder *D* is jointly trained using labeled and unlabeled data. The entire loss of the model consists of the fMRI loss of the encoder *E* and the Image loss (RGB and features loss) of the decoder *D*.

In a follow-up study, Gaziv et al. (2020) improved the reconstruction accuracy of BeliyEncDec by introducing a loss function based on the perceptual similarity measure (Zhang et al., 2018). To calculate perceptual similarity loss, the authors first extracted multilayer features from original and reconstructed images using VGG and then compared the extracted features layerwise. To distinguish it from BeliyEncDec, we refer to the framework proposed by Gaziv et al. (2020) as GazivEncDec.

### 3.2. Generative Methods

Generative models assume that the data is generated from some probability distribution *p*(**x**) and can be classified as implicit and explicit. Implicit models do not define the distribution of the data but instead specify a random sampling process with which to draw samples from *p*(**x**). Explicit models, on the other hand, explicitly define the probability density function, which is used to train the model.

#### 3.2.1. Generative Adversarial Network (GAN)

A class of implicitly defined generative models called Generative adversarial networks (GANs) received much attention due to their ability to produce realistic images (Goodfellow et al., 2014). In natural image reconstruction, GANs are widely used to learn the distribution of stimulus images. A GAN contains generator and discriminator networks. In the image generation task, the generator *G* takes a random noise vector **z** (generally sampled from a Gaussian distribution) and generates a fake sample *G*(**z**) with the same statistics as the training set images. During training, the generator's ability to generate realistic images continually improves until the discriminator is unable to distinguish the difference between a real sample and a generated fake one. GAN-based frameworks have several desirable properties compared to other generative methods. First, GANs do not require strong assumptions regarding the form of the output probability distribution. Second, adversarial training, which uses the discriminator, allows unsupervised training of the GAN (St-Yves and Naselaris, 2018). An illustration of GAN and details on GAN's loss function are provided in Supplementary Material.

To ensure that reconstructions resemble natural images Shen et al. (2019b) further modified their ShenDNN method by introducing a deep generator network (DGN) (Dosovitskiy and Brox, 2016). The framework is shown in Figure 3B. A DGN, pretrained on natural images using the GAN training process, is integrated with the DNN to produce realistic images, and the optimization is performed on the input space of the DGN. Thus, the reconstructed images are constrained to be in the subspace of the images generated by the DGN. We refer to these framework variations without and with DGN as ShenDNN and ShenDNN+DGN in future references.

**Figure 5:**
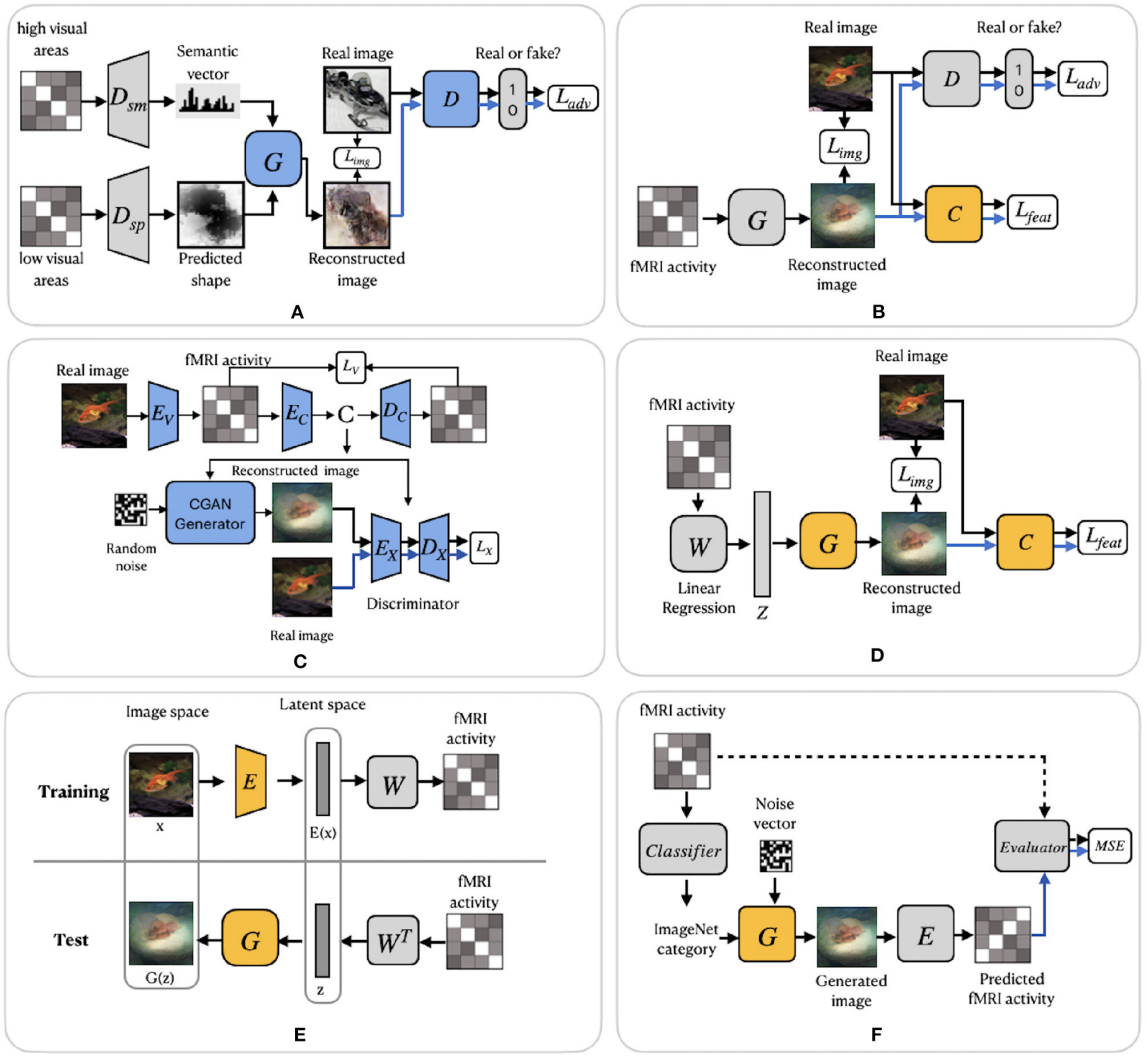
GAN-based frameworks. **(A)** FangSSGAN framework utilized a semantic decoder *D*_*sm*_ and a shape decoder *D*_*sp*_. **(B)** ShenGAN framework introduced a comparator network *C*. **(C)** StYvesEBGAN framework consists of three components trained independently: an encoding model *E*_*V*_, denoising autoencoder and *E*_*C*_–*D*_*C*_ and a conditional GAN. **(D)** SeeligerDCGAN framework based on deep convolutional GAN. **(E)** Framework proposed by Mozafari et al. (2020). **(F)** QiaoGAN-BVRM framework consists of four parts: a classifier, pretrained conditional generator, encoder, and evaluator network. For **(A–F)**, the pretrained components of the framework are highlighted in yellow. The blue color of the components indicates that they were trained using additional data. Images are from ImageNet dataset (Deng et al., 2009).

Similar to Shen et al. (2019b), Fang et al. (2020) based their work on the finding that visual features are hierarchically represented in the visual cortex. In the feature extraction step, the authors proposed two decoders, which extract shape and semantic representations from the lower and higher areas of visual cortex. The shape decoder *D*_*sp*_ is a linear model, and the semantic decoder *D*_*sm*_ has a DNN-based architecture (Figure 5A). In the image reconstruction step, the generator network *G* was trained with GAN using the extracted shape and semantic features as conditions for generating the images. We refer to this model as FangSSGAN, where SSGAN stands for the shape and semantic GAN. The generator *G* is a CNN-based network with an encoder–decoder structure (Ronneberger et al., 2015). To enhance reconstruction quality, approximately 1,200 additional images, different from those in the training/test set, were sampled from the ImageNet dataset to generate augmented data. These new images were used to generate shapes and category-average semantic features that were further passed into the GAN.

Another GAN-based model was proposed by Shen et al. (2019a). The end-to-end model directly learns the mapping from fMRI signals to reconstructed images without intermediate transformation or feature extraction (see Figure 5B). The framework, which we refer to as ShenGAN, was trained using three convolutional neural networks: a generator *G*, a comparator *C*, and a discriminator *D*. The generator *G* maps the fMRI data vector **v** to *G*(**v**), and a discriminator *D* distinguishes between reconstruction *G*(**v**) and the original image **x**. A comparator network *C* is pretrained on ImageNet (on image classification task) and used to compare the reconstruction *G*(**v**) with the original image **x** by calculating the perceptual loss (similarity in feature space). The combined loss function is a weighted sum of three terms: loss in image space, perceptual loss and adversarial loss.

The GAN-based methods described so far enhanced the quality of reconstruction by generating more natural-looking images. However, although GANs can generate new plausible samples matching the distribution of samples in the training dataset, they do not allow to control any characteristics of the generated data (Langr and Bok, 2019). To solve this issue, St-Yves and Naselaris (2018) implemented the conditional generation of images using a variation of GAN called the energy-based condition GAN or EBGAN (Zhao et al., 2017). In their framework, which we refer to as StYvesEBGAN, the authors first implement the encoding model *E*_*V*_ to learn the mapping from stimulus to fMRI, as shown in Figure 5C. In addition, StYvesEBGAN utilizes a denoising autoencoder to compress noisy high-dimensional fMRI representations into lower-dimensional representations. These lower-dimensional fMRI representations are further used as a condition vector for the GAN to reconstruct the stimuli. EBGAN is a more stable framework in terms of training than regular GANs. Instead of a binary classifier, it uses a deep autoencoder network as a discriminator. The authors observed that the reconstruction quality is highly dependent on the voxel denoising autoencoder, which produces a conditioning vector that results in the best reconstruction accuracy.

A group of studies by Seeliger et al. (2018), Mozafari et al. (2020), and Qiao et al. (2020) utilized GAN architecture with the assumption that there is a linear relationship between brain activity and the latent features of GAN. Similar to ShenDNN+DGN, these methods adopted the generator of a pretrained GAN as a natural image prior, which ensures that the reconstructed images follow similar distributions as natural images.

Seeliger et al. (2018) used a deep convolutional GAN (DCGAN) architecture (Radford et al., 2016), which introduced improvements by stacking convolutional and deconvolutional layers. The authors learn the direct linear mapping from the fMRI space to the latent space of GAN (see Figure 5D). For the natural stimuli image domain, the generator *G* was pretrained on down-sampled 64 × 64 converted-to-grayscale images from ImageNet (Chrabaszcz et al., 2017) and Microsoft COCO (Lin et al., 2014) datasets. For the handwritten character stimulus domain, DCGAN was pretrained on 15,000 handwritten characters from Maaten (2009) and Schomaker et al. (2000). Also, a pretrained comparator network *C*, based on AlexNet, was introduced as a feature-matching network to compute the feature loss *L*_*feat*_ across different layers. Overall, the loss is computed as a weighted sum of the pixelwise image loss *L*_*img*_ (MAE) and feature loss *L*_*feat*_. We refer to this framework as SeeligerDCGAN.

Mozafari et al. (2020) used a variation of GAN, called the BigBiGAN model (Donahue and Simonyan, 2019), which allowed the reconstruction of even more realistic images. The model generates high-level semantic information due to the BigBiGAN's latent space, which extracts high-level image details from fMRI data. We refer to this framework as MozafariBigBiGAN. The framework utilizes a pretrained encoder *E* that generates a latent space vector *E*(**x**) from the input image **x** and generator *G* that generates an image *G*(**z**) from the latent space vector **z** (see Figure 5E). During training, the authors computed the linear mapping *W* from latent vectors *E*(**x**) to fMRI activity using a general linear regression model. During the test stage, the linear mapping is inverted to compute the latent vectors **z** from the test fMRI activity.

The GAN-based Bayesian visual reconstruction model (GAN-BVRM) proposed by Qiao et al. (2020) aims to improve the quality of reconstructions from a limited dataset combination and, as the name suggests, uses the combination of GAN and Bayesian learning. From Bayesian perspective, a conditional distribution *p*(**v**|**x**) corresponds to an encoder which predicts fMRI activity **v** from the stimuli image **x**. On the other hand, an inverse conditional distribution *p*(**x**|**v**) corresponds to a decoder that reconstructs the stimuli from the fMRI activity. The goal of image reconstruction is to find the image that has the highest posterior probability *p*(**x**|**v**), given the fMRI activity. However, since the posterior distribution is hard to compute, Bayesian theorem is used to combine encoding model *p*(**v**|**x**) and image prior *p*(**x**) through *p*(**x**|**v**)∝*p*(**x**)*p*(**v**|**x**). The prior distribution *p*(**x**) reflects the predefined knowledge about natural images and is independent of the fMRI activity. The QiaoGAN-BVRM framework is shown in Figure 5F, and it consists of four parts: a classifier network, pretrained conditional generator *G*, encoder *E*, and evaluator network. First, a classifier decodes object categories from fMRI data, and then a conditional generator *G* of the BigGAN uses the decoded categories to generate natural images. The advantage of the pretrained generator is that it has already learned the data distribution from more than one million ImageNet natural images. Therefore, instead of searching the images one by one in a fixed image dataset (Naselaris et al., 2009), the generator can produce the optimal image reconstructions that best match with the fMRI activity via backpropagation. The generated images are passed to the encoder *E*, which predicts the corresponding fMRI activity. The proposed visual encoding model and the pre-trained generator of BigGAN do not interfere with each other, which helps to improve the fidelity and naturalness of reconstruction. The reconstruction accuracy is measured using an evaluator network, which computes the negative average mean squared error (MSE) between the predicted and actual fMRI activity. The reconstructions are obtained by iteratively updating the input noise vector to maximize the evaluator's score.

#### 3.2.2. VAE-GAN

The variational autoencoder (VAE) proposed by Kingma and Welling (2014) is an example of an explicit generative network and is a popular generative algorithm used in neural decoding. Similar to autoencoders, the VAE is composed of an encoder and a decoder. But rather than encoding a latent vector, VAE encodes a distribution over the latent space, making the generative process possible. Thus, the goal of VAE is to find a distribution of the latent variable **z**, which we can sample from **z**~*q*_ϕ_(**z**|**x**) to generate new image reconstructions ${\text{x}}^{\prime }\sim p_{\theta }\left(\text{x}|\text{z}\right)$. *q*_ϕ_(**z**|**x**) represents a probabilistic encoder, parameterized with ϕ, which embeds the input **x** into a latent representation **z**. *p*_θ_(**x**|**z**) represents a probabilistic decoder, parameterized with θ, which produces a distribution over the corresponding **x**. The details on VAE and its loss function are provided in Supplementary Material.

A hybrid model by Larsen et al. (2016) integrates both the VAE and GAN in a framework called VAE-GAN. VAE-GAN combines VAE to produce latent features and GAN discriminator, which learns to discriminate between fake and real images. In VAE-GAN, the VAE decoder and GAN generator are combined into one. The advantages of VAE-GAN are as follows. First, the GAN's adversarial loss enables generating visually more realistic images. Second, VAE-GAN achieves improved stability due to VAE-based optimization. This helps to avoid mode collapse inherent to GANs, which refers to a generator producing a limited subset of different outcomes (Ren et al., 2021; Xu et al., 2021).

**Figure 6:**
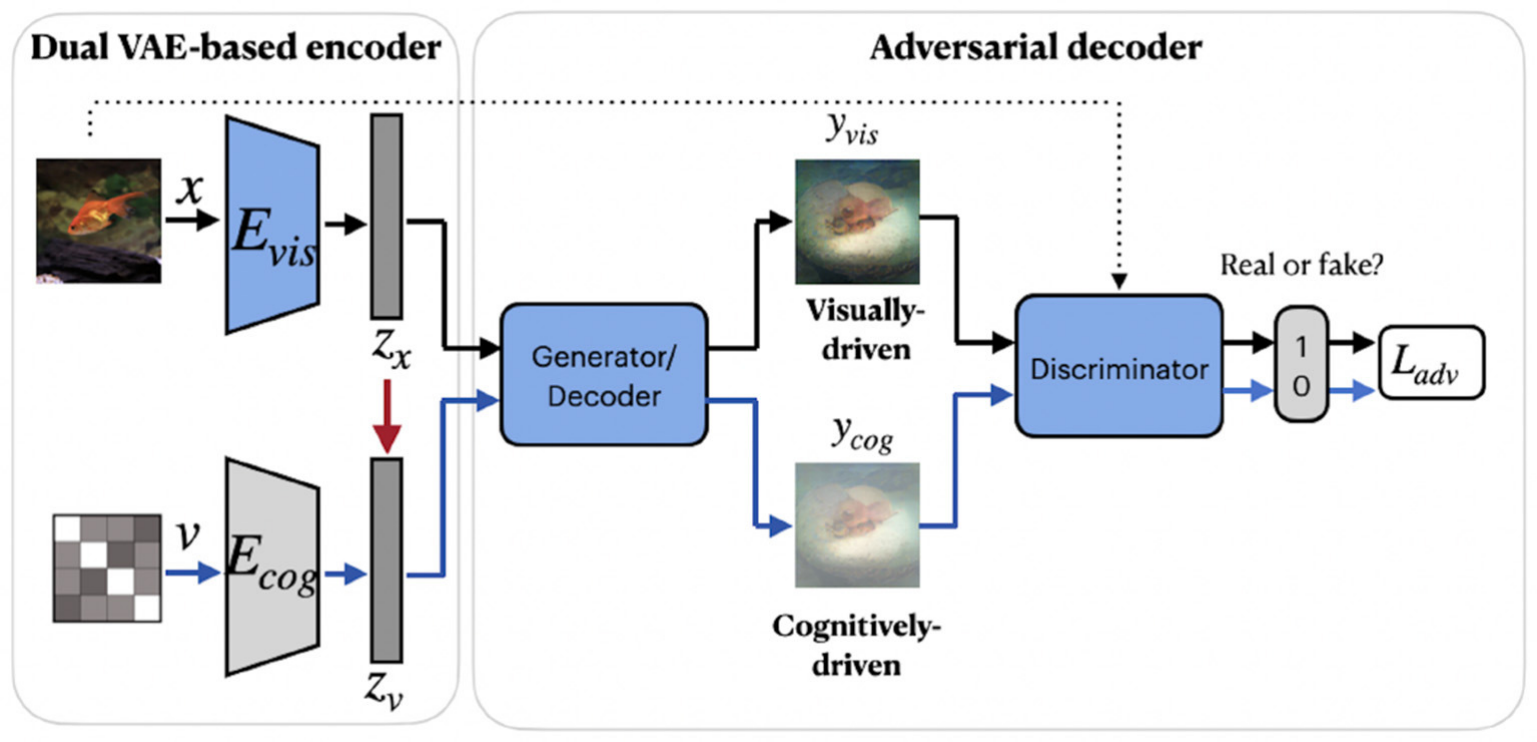
RenD-VAE/GAN framework consists of three main components: dual VAE-based encoder, adversarial decoder, and discriminator. The visual encoder *E*_*vis*_, cognitive encoder *E*_*cog*_, generator, and discriminator were used during the training. During testing, only a trained cognitive encoder and generator were used for the inference. The red arrow denotes the transfer of knowledge from the teacher network *E*_*vis*_ to the student network *E*_*cog*_. The components in blue denote training on external unlabeled natural images (without fMRI activity) from ImageNet, which do not overlap with images in the train/test set. Images are from ImageNet dataset (Deng et al., 2009).

A group of studies on reconstructing natural images from brain activity patterns, including Ren et al. (2021) and VanRullen and Reddy (2019), incorporated probabilistic inference using VAE-GAN. In a recent work by Ren et al. (2021), the authors presented a combined network called Dual-Variational Autoencoder/ Generative Adversarial Network (D-VAE/GAN). The framework, which we named RenD-VAE/GAN, consists of a dual VAE-based encoder and an adversarial decoder, as illustrated in Figure 6. Dual-VAE consists of two probabilistic encoders: visual *E*_*vis*_ and cognitive *E*_*cog*_, which encode stimuli images **x** and brain activity patterns **v** to corresponding latent representations **z_x_** and **z_v_**. The framework is trained in three sequential stages. In the first stage, visual stimuli images are used to train the visual encoder *E*_*vis*_, generator *G*, and discriminator *D*. *E*_*vis*_ learns the direct mapping from visual images into latent representations. Then, using output of *E*_*vis*_, the generator *G* is trained to predict the images and *D* is trained to discriminate the predicted images from real images. In the second stage, *E*_*cog*_ is trained to map high-dimensional fMRI signals to cognitive latent features. The generator *G* is fixed and *D* is trained to discriminate between the stimuli images produced in the first stage and the cognitively-driven reconstructions from cognitive latent features. This way, *E*_*cog*_ is forced to generate visual and cognitive latent representations similar to each other. In the last training stage, *E*_*cog*_ is fixed, whereas *G* and *D* are fine-tuned on fMRI signals to improve the accuracy of the generated images via cognitive latent representations. In this stage, *D* is trained to discriminate between real stimuli images and reconstructed images. During testing, only a trained cognitive encoder and generator were used for the inference. Since *E*_*vis*_ takes visual stimuli as input, its learned latent representations **z_x_** can guide *E*_*cog*_ to learn the latent representations **z_v_**. Thus, in the second training stage, the authors implement the concept of knowledge distillation by transferring knowledge from *E*_*vis*_ to *E*_*cog*_, which together represent the teacher and student networks (Hinton et al., 2015). The learned latent representation vectors significantly improve the reconstruction quality by capturing visual information, such as color, texture, object position, and attributes.

**Figure 7:**
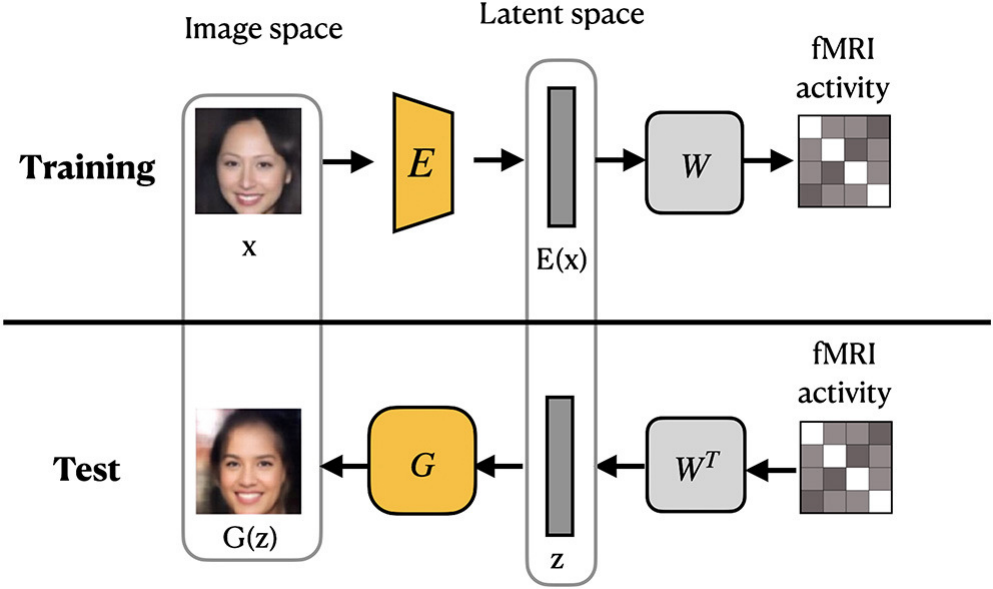
VanRullenVAE-GAN framework proposed by VanRullen and Reddy (2019). The encoder ***E*** maps a stimulus image onto the latent representation **z**. The generator *G* uses **z** to reconstruct the stimuli image. The pretrained components are shown in yellow. Images are from CelebA dataset (Liu et al., 2015).

VanRullen and Reddy (2019) utilized VAE network pretrained on CelebA dataset using GAN procedure to learn variational latent space. Similar to MozafariBigBiGAN framework, the authors learned a linear mapping between latent feature space and fMRI patterns, rather than using probabilistic inference (Güçlütürk et al., 2017). In the training stage, the pretrained encoder from VAE-GAN is fixed and the linear mapping between latent feature space and fMRI patterns is learned. For the test stage, fMRI patterns are first translated into VAE latent codes via inverse mapping, and then these codes are used to reconstruct the faces. The latent space of a VAE is a variational layer that provides a meaningful description of each image and can represent faces and facial features as linear combinations of each other. Owing to the training objective of the VAE, the points which appear close in this space are mapped onto similar face images, which are always visually plausible. Therefore, the VAE's latent space ensures that the brain decoding becomes more robust mapping errors. As a result, the produced reconstructions from VAE-GAN appear to be more realistic and closer to the original stimuli images. This method not only allows the reconstruction of naturally looking faces but also decodes face gender. In terms of architecture, the framework, which we refer to as VanRullenVAE-GAN, consists of three networks, as shown in Figure 7.

## 4. Reconstruction Evaluation

**Figure 8:**
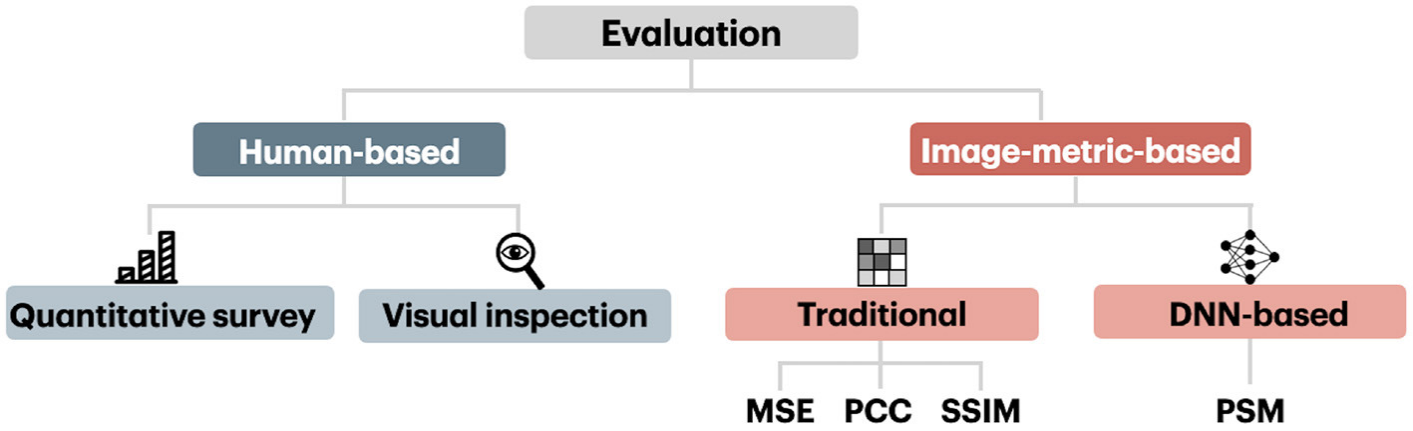
Image-metric-based and human-based evaluation.

The evaluation of reconstruction methods is based on human-based and image metrics, which we schematically present in Figure 8. We first present human-based and image metrics and then describe the differences in image comparison settings.

### 4.1. Human-Based Evaluation

The intuitive method of measuring the quality of reconstruction in natural image reconstruction task is by employing human evaluators. Human-based evaluation can be conducted quantitatively and qualitatively through visual inspection.

For quantitative human-based assessment, a behavioral study involving human subjects is conducted. In this study the reconstructed image is compared to the original or several candidate images, containing the original image. From the given candidate images, subjects are instructed to choose the one that appears to have a higher resemblance to the original. Such behavioral studies can be conducted by employing human evaluators or using Amazon Mechanical Turk^6^ (Seeliger et al., 2018; Gaziv et al., 2020).

**Figure 9:**
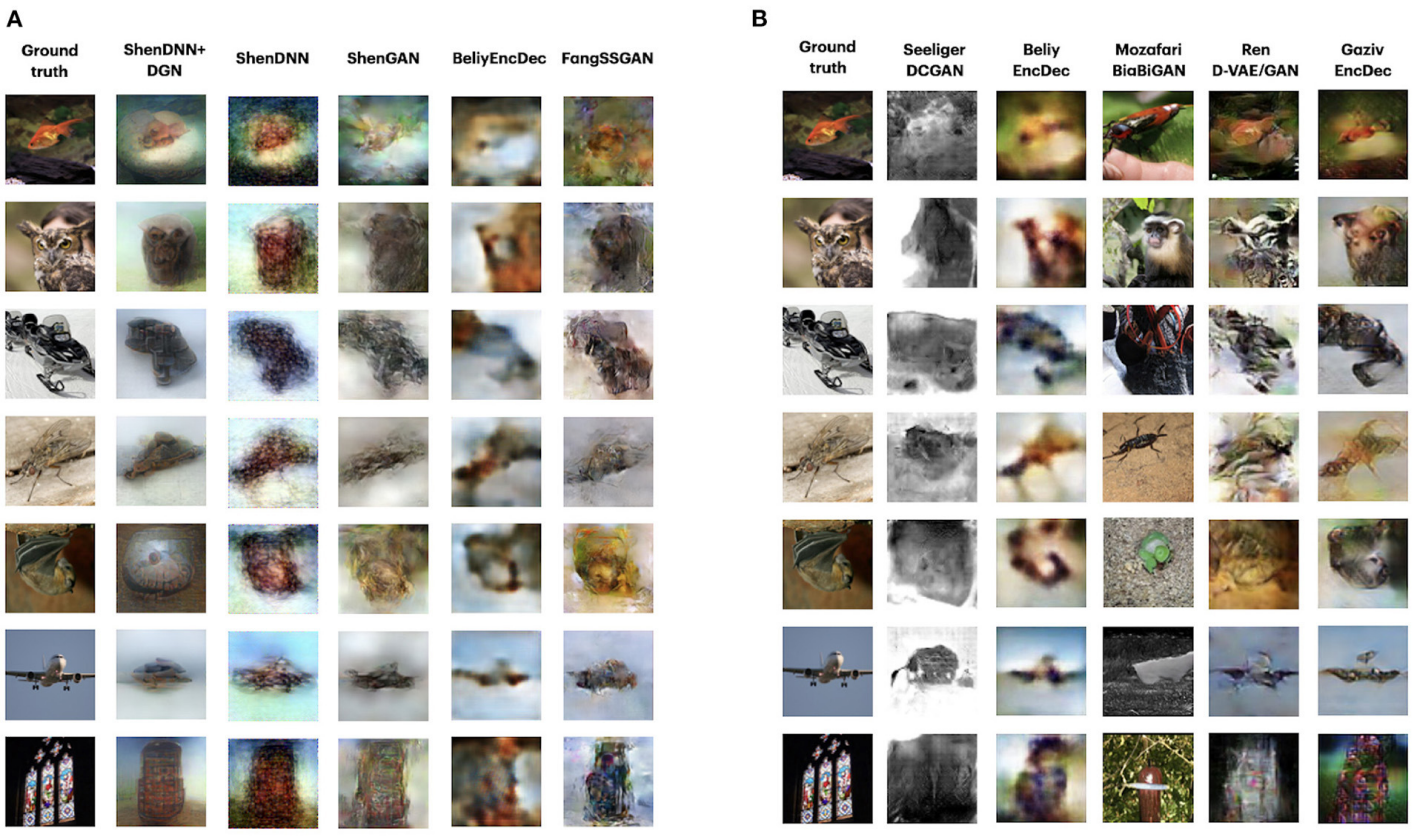
**(A)** Visual comparison of methods on Deep Image Reconstruction dataset for subject 1. The reconstructions for all methods except for Fang et al. (2020) are obtained by reproducing the experiments. For **(A,B)**, the stimulus images are shown in the first column. The corresponding reconstructed images from each method are shown in the subsequent columns. **(B)** Visual comparison of the methods on the GOD dataset. Due to the unavailability of complete reconstruction data for GOD, visual reconstructions correspond to the same image stimuli but different subjects. For BeliyEncDec and GazivEncDec, we present the reconstruction for subject 3. The reconstructions for all methods are provided by the authors or reported in the original papers. SeeligerDCGAN uses the average of the stimuli representations for the three subjects. Images are from ImageNet dataset (Deng et al., 2009).

Owing to the additional time and human input required for human-based evaluation, several recent studies omit quantitative human evaluation in favor of qualitative visual inspections. For visual comparison, the set of original images and their reconstructions from different reconstruction methods are juxtaposed for ease of comparison (see Figures 9A,B). Reconstructions are usually compared in terms of image sharpness/blurriness, matching shapes, colors, and low/high-level details. Many recent works focus on emphasizing the “naturalness” of their reconstructions, despite the reconstructions deviating significantly from the actual images in terms of the object category (see reconstructions in column 4 in Figure 9B for example).

Although human-based evaluation is a more reliable measurement of the quality of the reconstructed image, it suffers from the following limitations. First, human-based evaluation is time consuming and expensive because the process requires a well-designed evaluation study and the recruitment of human subjects. Second, the results can be heavily affected by subjects' physical and emotional conditions or external conditions, such as lighting or image display (Wang et al., 2004; Rolls, 2012). Table 3 shows that only several studies conducted the quantitative human-based evaluation.

**Table 3:** Comparison of methods in terms of the used evaluation metrics.

	**Human-based metrics**		**Image metrics**	
**References**	**Quantitative survey**	**Visual inspection**	**Traditional**	**PSM**
Seeliger et al. (2018)	✓	✓	✗	✗
Shen et al. (2019b)	✓	✓	Pairwise PCC	✗
Shen et al. (2019a)	✓	✓	Pairwise PCC Pairwise SSIM	✗
Beliy et al. (2019)	✗	✓	2,5,10-way PCC	✗
Gaziv et al. (2020)	✓	✓	✗	2,5,10-way PSM
Qiao et al. (2020)	✗	✓	PCC SSIM	AlexNet
Fang et al. (2020)	✗	✓	Pairwise PCC	✗
Mozafari et al. (2020)	✗	✓	Pairwise PCC Pix-Comp (2-way PCC)	Inception-V3
Ren et al. (2021)	✓	✓	Linear correlation SSIM 2,5,10-way PCC	✗

*PCC stands for the Pearson correlation coefficient*.

### 4.2. Image-Metric-Based Evaluation

As an alternative to human-based evaluation, image-metric-based evaluation is used to accurately and automatically assess image reconstruction quality. The use of image metrics for evaluation is more practical, and unlike human-based assessment, is unbiased toward external factors. However, the image-metric-based evaluation can provide only an approximation of the visual comparison mechanism inherent to a human subject, and thus are far from being perfect (Wang et al., 2004).

Nowadays, there exist various image metrics that can compare images at different levels of perceptual representation. Image metrics used in the visual decoding literature can be categorized into traditional metrics that capture low-level perceptual similarities and more recent ones that capture high-level perceptual similarity. The conventional metrics, which include the mean squared error (MSE), pixelwise Pearson correlation coefficient (PCC), structural similarity index (SSIM), and their variants, are computed in pixel space and capture low-level perceptual similarity. The metric that captures high-level perceptual similarity relies on multilevel feature extraction from DNN and can compare images at a higher level of perceptual representation. The high-level metric we considered here is called Perceptual Similarity Metric (PSM).

#### 4.2.1. MSE

MSE is the simplest traditional metric for assessing image reconstruction quality. Given *x*_*i*_ and *y*_*i*_, which are the flattened one-dimensional representations of the original and the reconstructed images, the MSE estimated over *N* samples is computed as


(1) $$\begin{array}{r} MSE=\frac{1}{N}\overset{N}{\underset{i=1}{\sum }}{\left(x_{i}-y_{i}\right)}^{2}. \end{array}$$

Several characteristics of MSE, including simplicity of implementation and fast computation, make it a widely used performance metric in signal processing. However, MSE shows poor correspondence to human visual perception, due to some of the underlying assumptions: MSE is independent of the spatial relationship between image pixels and considers each of them to be equally important (Wang et al., 2004).

#### 4.2.2. PCC

PCC is widely used in statistical analysis to measure the linear relationship between two variables. The following equation is used to compute the pixelwise Pearson correlation between the flattened 1-D representations of the original image *x* and the reconstructed image *y*:


(2) $$\begin{array}{r} PCC\left(x,y\right)=\frac{\sum \left(x-\mu_{x}\right)\left(y-\mu_{y}\right)}{\sqrt{\sum {\left(x-\mu_{x}\right)}^{2}\sum {\left(y-\mu_{y}\right)}^{2}}}, \end{array}$$

where μ_*x*_ and μ_*y*_ are the mean intensities of the flattened one-dimensional vectors *x* and *y*, respectively. PCC is the most common metric used across the surveyed works (see Table 3), with slight variations in naming and implementation: Pairwise PCC (Shen et al., 2019a,b), pixel correlation (Ren et al., 2021), Pix-Comp (Mozafari et al., 2020), and n-way PCC (Beliy et al., 2019). The limitation of PCC is its sensitivity to changes in the edge intensity or edge misalignment. Thus, the metric tends to assign higher scores to blurry images than to images with distinguishable but misaligned shapes (Beliy et al., 2019).

#### 4.2.3. SSIM

SSIM is widely used image similarity metric that captures structural information from images. Wang et al. proposed SSIM as a quality assessment metric that resembles the characteristics of the human visual perception (Wang et al., 2004). Unlike PCC, which treats each pixel of the image independently, SSIM measures the similarity of spatially close pixels between the reconstructed and original images. Given two images, SSIM is computed as a weighted combination of three comparative measures: luminance, contrast, and structure. Assuming an equal contribution of each measure, the SSIM is first computed locally between the corresponding windows *p* and *q* of images *x* and *y*:


(3) $$\begin{array}{r} SSIM\left(p,q\right)=\frac{\left(2\mu_{p}\mu_{q}+C_{1}\right)\left(2\sigma_{pq}+C_{2}\right)}{\left(\mu_{p}^{2}+\mu_{q}^{2}+C_{1}\right)\left(\sigma_{p}^{2}+\sigma_{q}^{2}+C_{2}\right)}, \end{array}$$

where μ_*p*_ and μ_*q*_ are the mean intensity values of *p* and *q*, respectively; $\sigma_{p}^{2}$ and $\sigma_{q}^{2}$ are the variances of *p* and *q*, respectively; σ_*pq*_ is the covariance of *p* and *q*, and *C*_1_ and *C*_2_ are constants that ensure stability when the denominator is close to zero. The global SSIM score is computed as the average of all *M* local SSIM scores:


(4) $$\begin{array}{r} SSIM\left(x,y\right)=\overset{M}{\underset{i=1}{\sum }}SSIM\left(p_{i},q_{i}\right). \end{array}$$

#### 4.2.4. PSM

Despite the wide adoption of SSIM as a perceptual metric, it compares poorly with many characteristics of human perception (Zhang et al., 2018). Several studies, including Güçlütürk et al. (2017), Qiao et al. (2018), Mozafari et al. (2020), and Gaziv et al. (2020), emphasize the importance of higher-level perceptual similarity over lower-level metrics in evaluation because of the better correspondence of higher-level perceptual similarity to human perceptual judgments (Zhang et al., 2018).

As the general principle, a CNN is used for extracting hierarchical multilayer features of input images, which are further compared across layers using a distance metric of choice. However, the definition and implementation of the perceptual similarity metric in terms of the distance metric or feature extraction network vary across studies. For example, Qiao et al. (2020) utilized five convolutional layers of the AlexNet (Krizhevsky et al., 2012) to extract hierarchical features. The other study by Mozafari et al. (2020), proposed a high-level similarity measure, which measures perceptual similarity based on the output of only the last layer of Inception-V3 (Szegedy et al., 2016). Finally, Gaziv et al. (2020) used the PSM definition proposed in (Zhang et al., 2018) with the pretrained AlexNet with linear calibration. Following Gaziv et al. (2020), we provide a PSM definition by Zhang et al. (2018) in the following equation:


(5) $$d\left(x,y\right)=\sum_{l}\frac{1}{H_{l}W_{l}}\sum_{h,w}{\left\|w_{l}⊙\left(f_{x}^{l}-f_{y}^{l}\right)\right\|}_{2}^{2},$$

where *d*(*x, y*) is the distance between the original image *x* and the reconstructed image *y*. $f_{x}^{l},f_{y}^{l}$ represent layerwise activations normalized across channels for layer *l*. The activations are scaled channelwise by vector $w_{l}\in {\mathbb{R}}^{C_{l}}$, spatially averaged, and summed layerwise.

Note that the underlying CNN model used for computing the PSM should be selected cautiously. Because many studies use pretrained CNN models, it is important to avoid using the same model for both training and evaluation, which may lead to a potential bias in evaluation. For example, several methods, including Shen et al. (2019b) and Beliy et al. (2019), used VGG-19 (Simonyan and Zisserman, 2015) for pretraining. Therefore, the VGG-19 model should not be used for evaluation, as the objective of evaluation and optimization functions would be the same, and the evaluation would produce a higher similarity between original and reconstructed images.

### 4.3. Image Comparison Setting

We describe three image comparison settings existing in literature: (1) one-to-one comparison, (2) pairwise comparison, and (3) *n*-way comparison. Each of these comparison settings can work with any image or human-based metric of choice.

**One-to-one** is the simplest comparison setting which computes the similarity score of a reconstruction against ground truth using the given metric, for example, MSE or PCC. However, the absolute values of qualitative metrics computed only on a single pair of original and reconstructed images are challenging to interpret (Beliy et al., 2019). Therefore, pairwise similarity and *n*-way identification are often used to measure the reconstruction quality across the dataset.

**Pairwise comparison** analysis is performed by comparing a reconstructed image with two candidate images: the ground-truth image and the image selected from the remaining set, resulting in a total of *N*(*N*−1) comparisons:


(6) $$\begin{array}{r} score=\frac{1}{N\left(N-1\right)}\overset{N}{\underset{i=1}{\sum }}\overset{N}{\underset{\begin{array}{c} j=1\\ j\neq i \end{array}}{\sum }}\sigma \left(m\left(y_{i},x_{i}\right),m\left(y_{i},x_{j}\right)\right), \end{array}$$

where *m* is the metric of interest and


(7) $$\sigma (a,b)=\{\begin{array}{ll} 1 & a>b\\ 0 & \text{ otherwise } \end{array}$$

The trial is considered correct if the metric score of the reconstructed image with the corresponding stimulus image is higher than that with the non-relevant stimulus image. For metrics that imply that the lower, the better (such as MSE), the expression in the equation 7 is modified to find the smallest value. Finally, the percentage of total correct trials is computed as the ratio of correct trials among all trials (Beliy et al., 2019; Shen et al., 2019a,b). The chance-level accuracy is 50%.

In ***n*-way identification** each reconstructed image is compared to *n* randomly selected candidate images, including the ground truth. Several studies, including Beliy et al. (2019) and Ren et al. (2021), used *n* = 2, 5, 10 for the *n*-way identification accuracy computed using PCC. In a more recent work, Gaziv et al. (2020) report *n* = 2, 5, 10, 50-way identification accuracy based on PSM. An addition source of confusion is the absence of naming conventions: Ren et al. (2021) and Mozafari et al. (2020) referred to *n*-way identification accuracy computed with PCC as Pixel Correlation and pix-Comp, respectively.

## 5. Fair Comparison Across the Methods

For fair comparison of the existing methods, we chose those that satisfied one of the following criteria: (1) the availability of the complete code for reproducing the results and (2) the availability of reconstructed images for running the evaluation. This allowed us to compare five state-of-the-art methods on the DIR dataset, both visually (section 5.3) and quantitatively (section 5.4). For the GOD, because of the lack of a complete set of reconstructions for the chosen methods, we only present a visual comparison in section 5.3. Visual comparison for vim-1 datasets is provided in Supplementary Material.

Our analysis of recent works on natural image reconstruction reveals that only a few comply with good machine learning practices regarding the fairness of evaluation. Unfair evaluation can be reflected in the comparison across different datasets, selecting specific subjects in reporting the results, and discrepancies in using the evaluation metrics. This motivated us to perform a rigorous empirical evaluation of the methods, i.e., *cross-subject* evaluation across *common* metrics using a *common* dataset.

### 5.1. Evaluation on a Common Dataset

To standardize the objective evaluation process, we perform the quantitative assessment on the DIR dataset for methods that we found to be reproducible, that is, ShenDNN, ShenDNN+DGN, ShenGAN, and BeliyEncDec. For FangSSGAN, we ran an evaluation based on the reconstructions provided by the authors.

It is important to distinguish the five-subject GOD dataset (Horikawa and Kamitani, 2017) from the three-subject DIR dataset (Shen et al., 2019b), which uses the same stimuli images but is quite different in terms of the image presentation experiment and characteristics of fMRI activity data. Our choice of the DIR as a common natural image dataset is due to the following reasons. First, unlike the similar GOD dataset, DIR was acquired specifically for the natural image reconstruction task and contains a larger number of training samples due to increased number of repeats in image presentation experiment. In addition, this dataset might be of interest for studying the generalizability of natural image reconstruction methods to artificial shapes, which we describe in detail in Supplementary Material.

When training ShenDNN, ShenDNN+DGN, and BeliyEncDec on the DIR, we used the original training settings. For ShenGAN, we used the pretrained model provided by the authors. To maximize the amount of training data, each presented stimulus sample was treated as an individual sample (Shen et al., 2019a). For reconstruction, we averaged the test fMRI activations across trials corresponding to the same stimulus to increase the signal-to-noise ratio. This resulted in 6,000 training and 50 test fMRI-image samples. Note that BeliyEncDec was initially implemented for GOD dataset. For BeliyEncDec, averaging both training and test fMRI samples across the repeats resulted in the best performance. This confirms with the authors' observation that an increased number of fMRI repeats results in improved reconstruction (Beliy et al., 2019). Additionally, we normalized training fMRI vectors to have a zero mean and unit standard deviation. The mean and standard deviation of the training fMRI data were used to normalize the test fMRI data.

### 5.2. Evaluation Across Common Metrics

We perform the evaluation on natural images from the DIR based on MSE, PCC, SSIM, and PSM metrics described in section 4. We notice that there is no consensus among recent works on a standard set of evaluation metrics (see Table 3). Moreover, several studies introduce new evaluation metrics or use variations of existing metrics, potentially more favorable for their results. In contrast, we present an evaluation of the methods across all the image metrics used in the related methods.

It is also important to note that different methods generate output images of various sizes due to memory restrictions and variations in the pretrained model (we refer to Supplementary Material for details on output image resolutions). The evaluation metrics can be sensitive to the size of the image and the choice of upscaling or downscaling algorithms. For fairness, we rescaled the reconstructions for the DIR to the common size and use a bicubic algorithm for image resizing. We evaluated the reconstructed images using a resolution of 256 × 256 pixels, which is the highest among the chosen methods. For methods with a lower reconstruction image size, we applied image upscaling.

### 5.3. Visual Comparison Results

Figure 9A shows the reconstructions of sample stimuli images from the test set, corresponding to subject 1 from DIR dataset. The reconstructions from all methods show a close resemblance to the original images in terms of the object shape and position. GAN-based methods, i.e., ShenDNN+DGN and ShenGAN, produce sharper and smoother-looking images but in some cases render semantic details absent in the original stimuli (which is confirmed by lower pixelwise MSE and PCC scores). Reconstructions by FangSSGAN are also natural looking and close to real images in terms of shape and color. This is attributed to using a generator network conditioned on both shape and semantic information, which preserves low-level features, such as texture or shape. Reconstruction by non-GAN^7^ BeliyEncDec are blurry but accurately capture the shape of the stimuli objects.

In addition, we present the reconstructions for GOD dataset in Figure 9B. Similar to DIR dataset, the GAN-based methods MozafariBigBiGAN and RenD-VAE/GAN produce the most natural-looking images. Visually, MozafariBigBiGAN outperforms other methods in terms of naturalness. However, this comes at the cost of rendering object categories and details different from those presented in the original stimuli. We identified GazivEncDec and RenD-VAE/GAN as performing relatively better on the reconstruction of shape and color. GazivEncDec is superior in reconstructing high-level details of the image, including shape and background. RenD-VAE/GAN visually outperforms other methods for the reconstruction of color, background, and lower-level details. For GazivEncDec, a significant improvement in the reconstruction accuracy was achieved owing to the introduced perceptual similarity loss. According to Ren et al. (2021), the key factors boosting the reconstruction quality of the RenD-VAE/GAN include the VAE-GAN architecture instead of the standard conditional GAN and visual-feature guidance implemented via GAN-based knowledge distillation. In SeeligerDCGAN and BeliyEncDec, the reconstructions are blurry, which could be due to the use of pixelwise MSE loss (Seeliger et al., 2018).

**Figure 10:**
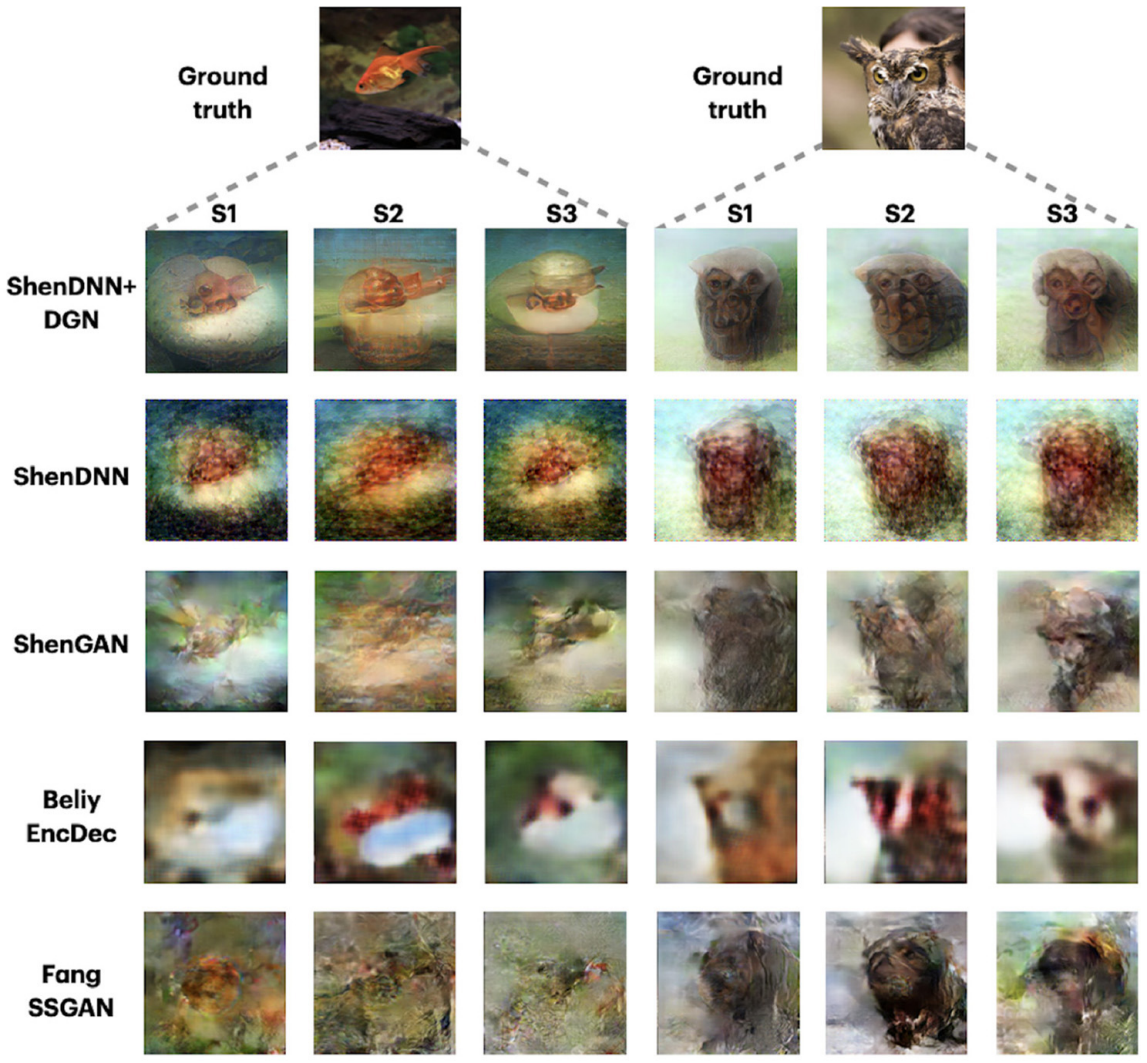
Reconstructions for two images across three subjects from DIR dataset. Images are from ImageNet dataset (Deng et al., 2009).

Since the DIR dataset comprises three-subject data, we additionally show the reconstructions across the methods corresponding to three different subjects in Figure 10. The reconstructions are shown for the two natural image stimuli. Depending on the subject, the reconstructions by different methods show varying degrees of resemblance to the original stimuli. For example, the reconstructions from ShenDNN+DGN, ShenGAN, and BeliyEncDec are visually better for subject 1, whereas, in reconstructions by other methods, neither color nor shape was preserved. This shows that the selection of a subject in reporting results can lead to a biased evaluation.

### 5.4. Quantitative Comparison Results on Natural Images From DIR

**Figure 11:**
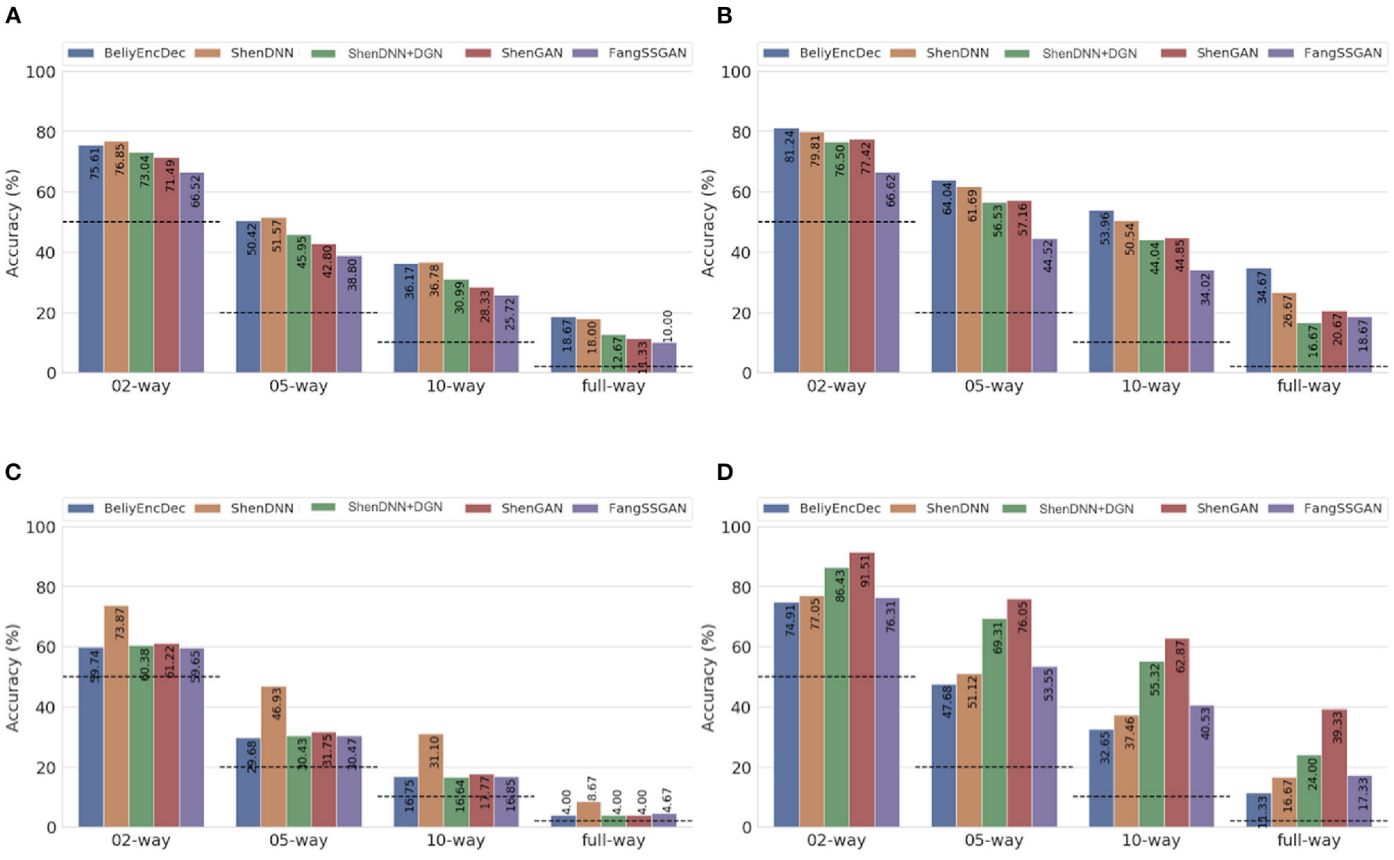
Average *n*-way accuracy results computed across subjects using **(A)** MSE, **(B)** PCC, **(C)** SSIM, and **(D)** PSM metrics on natural images from the DIR. The horizontal dashed lines indicate the chance level for each metric. The full-way comparison corresponds to using all the images in the test set, that is, 50 natural images.

To eliminate the bias of selecting a specific subject for evaluation, we present both subject-specific and cross-subject average results across multiple metrics on natural images from DIR. For comprehensive evaluation, we use three comparison settings described in section 4: (1) one-to-one comparison; (2) pairwise comparison; and (3) *n*-way comparison. The pairwise evaluation results for natural images across the metrics are shown in Table 4. The *n*-way scores for natural images are presented in Figure 11. We find that one-to-one results are not well-suited for cross-method comparison. We therefore present a one-to-one comparison in Supplementary Material. The quantitative evaluation of methods is presented based on low-level MSE, PCC, and SSIM metrics first, followed by a comparison using a high-level PSM metric.

**Table 4:** Pairwise evaluation across the methods on natural images from the DIR dataset.

**Subject**	**Method**	**MSE ↑**	**PCC↑**	**SSIM ↑**	**PSM ↑**
S1	ShenDNN	75.80	80.69	**75.59**	77.67
	ShenDNN+DGN	74.53	78.98	61.27	86.61
	ShenGAN	71.67	79.06	62.08	**92.33**
	BeliyEncDec	**76.94**	**86.08**	59.67	73.14
	FangSSGAN	67.71	67.18	60.37	76.12
S2	ShenDNN	**74.98**	**77.27**	**70.82**	77.14
	ShenDNN+DGN	70.78	75.43	59.55	86.41
	ShenGAN	68.65	74.20	59.51	**90.41**
	BeliyEncDec	71.18	76.20	58.94	75.22
	FangSSGAN	64.00	66.24	58.69	**73.71**
S3	ShenDNN	**79.71**	**81.47**	**75.06**	76.20
	ShenDNN+DGN	73.59	75.02	60.24	86.20
	ShenGAN	74.12	78.98	62.08	**91.88**
	BeliyEncDec	78.61	**81.47**	60.53	76.20
	FangSSGAN	67.88	66.45	59.96	79.02
Average result	ShenDNN	**76.83 ± 2.53**	79.81 ± 2.24	**73.82 ± 2.62**	77.01 ± 0.74
	ShenDNN+DGN	72.97 ± 1.95	76.48 ± 2.18	60.35 ± 0.86	86.41 ± 0.20
	ShenGAN	71.48 ± 2.74	77.41 ± 2.78	61.22 ± 1.48	**91.54 ± 1.00**
	BeliyEncDec	75.58 ± 3.90	**81.25 ± 4.94**	59.71 ± 0.80	74.86 ± 1.56
	FangSSGAN	66.53 ± 2.19	66.63 ± 0.49	59.67 ± 0.87	76.29 ± 2.66

*The best results are presented in bold*.

*↑indicates the higher the better*.

#### 5.4.1. Performance Using Low-Level Metrics

Based on the average results across the subjects shown in Table 4 and Figures 11A–C, two non-GAN methods lead on low-level metrics, namely ShenDNN and BeliyEncDec. Together, they outperform other baselines across three low-level pairwise metrics (i.e., pairwise MSE, pairwise PCC, and pairwise SSIM) as well as across *n*-way MSE and PPC metrics. The high performance of BeliyEncDec on low-level metrics can be attributed to efficient low-level feature extraction via encoder–decoder architecture and to the self-supervised training procedure with the extended set of unlabeled images and fMRI data. The high performance of ShenDNN on low-level metrics is potentially due to iterative pixel-level optimization of the reconstructed image.

#### 5.4.2. Performance Using the High-Level PSM Metric

Additionally, we compare the selected methods on the PSM implemented using AlexNet. From Table 4 and Figure 11D, we can see that ShenGAN performs the best on the high-level PSM metric, computed in a pairwise, and *n*-way manner across the subjects and on averages. Overall, GAN-based methods, including ShenGAN, ShenDNN+DGN, and FangSSGAN, which were reported to produce more natural-looking images, achieved the top three average results in most cases. This supports the motivation to utilize PSM for measuring high-level visual similarity in images, especially for GAN-based methods whose strength lies in reconstructing high-level visual features and more natural-looking images. We attribute the improved performance of the three methods to using a pretrained generator network and the superior performance of ShenGAN and ShenDNN+DGN to the use of multilayer DNN features for computing the multi-level feature loss. Notably, the performance of all metrics reduces as the *n*-way comparison becomes increasingly harder with an increasing number of samples being used in the comparison.

## 6. Discussion

Even with a relatively small number of the available open-source reconstruction frameworks, the visual and quantitative results presented in this work can give a general idea of which architectural solution, benchmark dataset, or evaluation framework can be chosen for experimental purposes.

Depending on the target of the reconstruction task, it is vital to consider the trade-off between the “naturalness” and the fidelity of the reconstruction. Generative methods rely on GAN or VAE-GAN-based architectures to produce the most natural-looking images and correspondingly higher PSM scores. However, they often require either external data for training or the use of pretrained network components. The availability of external image datasets for training becomes a significant factor for generating high-quality images for GAN. Most importantly, the methods that perform best at “naturalness” do not guarantee that the object categories of reconstruction will always match those of the original images, as in the case of MozafariBigBiGAN. Other non-generative methods developed for natural image reconstruction, such as BeliyEncDec or ShenDNN, do not produce realistic-looking images. However, whenever the fidelity of the reconstructions is preferable, these non-generative methods should be considered, as they exhibit closer similarity to the original images in terms of low-level features, which are supported both visually and quantitatively.

In this work we advocate the fairness in reconstruction evaluation procedure and discuss several criteria which should be standardized across the methods. At the same time, we believe that the evaluation procedure presented in this work can be further improved in the following ways.

**Availability of large-scale imaging data**. The primary challenge for current deep learning-based methods is that they are required to resolve the limitation of small-size fMRI data. Nowadays, the lack of training data is compensated by pretraining DNN components on external image data (Beliy et al., 2019; Shen et al., 2019a,b), self-supervision on additional image-fMRI pairs (Beliy et al., 2019; Gaziv et al., 2020) and generation of new surrogate fMRI via pretrained encoding models (St-Yves and Naselaris, 2018). Several brain imaging datasets are available for reconstruction tasks. However, larger scale datasets are still required. The availability of large-scale imaging data may improve current state-of-the-art results and foster research on reconstructing more complex and dynamic visual perception, including imagined images or videos. This, in turn, may lead to broader adoption of the proposed frameworks for real-world purposes.

**Developing new computational similarity metrics corresponding to human vision**. While some of the deep learning methods achieve encouraging results on high-level perceptual similarity metrics, an open question about the correspondence of these computer-based metrics to human vision remains. Because most accuracy evaluation metrics are oriented toward computer vision tasks, they often fail to capture the characteristics of human vision. Research in this direction might further advance natural image reconstruction by developing more advanced learning and evaluation metrics.

## 7. Conclusion

This paper presented an overview of state-of-the-art methods for natural image reconstruction task using deep learning. These methods were compared on multiple scales, including architectural design, benchmark datasets, and evaluation metrics. We highlighted several ambiguities with the existing evaluation and presented a standardized empirical assessment of the methods. This evaluation procedure can help researchers in performing a more comprehensive comparative analysis and elucidating the reason for the effectiveness of their method. We hope this study will serve as a foundation for future research on natural image reconstruction targeting fair and rigorous comparisons.

## Data Availability Statement

The original contributions presented in the study are included in the article/Supplementary Material, further inquiries can be directed to the corresponding author/s.

## Author Contributions

ZR: conceptualization, methodology, software, writing, and evaluation. QJ: software, evaluation, and writing—review and editing. XL: conceptualization, data curation, and writing—original draft preparation. TM: supervision and writing—review and editing. All authors contributed to the article and approved the submitted version.

## Funding

This work was partly supported by JST CREST (Grant Number JPMJCR1687), JSPS Grant-in-Aid for Scientific Research (Grant Number 21K12042, 17H01785), and the New Energy and Industrial Technology Development Organization (Grant Number JPNP20006).

## Conflict of Interest

The authors declare that the research was conducted in the absence of any commercial or financial relationships that could be construed as a potential conflict of interest.

## Publisher's Note

All claims expressed in this article are solely those of the authors and do not necessarily represent those of their affiliated organizations, or those of the publisher, the editors and the reviewers. Any product that may be evaluated in this article, or claim that may be made by its manufacturer, is not guaranteed or endorsed by the publisher.

## Footnotes

^1^The fMRI dataset is available at https://openneuro.org/datasets/ds001761.

^2^The dataset is available at http://crcns.org/data-sets/vc/vim-1.

^3^ https://www2.eecs.berkeley.edu/Research/Projects/CS/vision/grouping/segbench/

^4^The dataset can be acquired from http://brainliner.jp/data/brainliner/Generic_Object_Decoding.

^5^ https://openneuro.org/datasets/ds001506/versions/1.3.1

^6^ www.mturk.com

^7^By “non-GAN” methods, we mean the models that do not take advantage of the GAN training procedure.
