# Supplementary material for: Natural Image Reconstruction From fMRI Using Deep Learning: A Survey
Source: Front Neurosci. 2021 Dec 20;15:795488. doi: 10.3389/fnins.2021.795488 (PMC8722107; doi:10.3389/fnins.2021.795488)
Supplement: Supplementary file 1 [file Presentation_1.pdf]

Supplementary Material

1 OVERVIEW OF DEEP LEARNING METHODS

Encoder–decoder methods. In computer vision, encoder–decoder models are multilayer models that incorporate convolutional layers. Encoder–decoder models are widely used in image-to-image translation (Isola et al., 2017) and sequence-to-sequence models (Cho et al., 2014). They learn the mapping from an input domain to an output domain via a two-stage architecture: an encoder E that compresses the input to the latent space representation $\mathbf{z} = E(\mathbf{x})$ and a decoder D that produces the output from the latent representation $\mathbf{y} = D(\mathbf{z})$ (see Figure S1 A) (Minaee et al., 2021). The model is trained to minimize the reconstruction error, which is the difference between the reconstructed image and ground-truth image.

Generative Adversarial Network (GAN). A GAN, shown in Figure S1 B, contains generator and discriminator networks. In the image generation task, the generator G takes a random noise vector $\mathbf{z}$ (generally sampled from a Gaussian distribution) and generates a fake sample $G(\mathbf{z})$ with the same statistics as the training set images. The task of the discriminator network D is to distinguish the generated fake sample $G(\mathbf{z})$ from the real sample $\mathbf{x}$ by maximizing the probability $D(\mathbf{x})$ and minimizing $D(G(\mathbf{z}))$. This learning process is formulated as a zero-sum game with the following mini–max loss (Goodfellow et al., 2014):

$$E_{\mathbf{x}}[\log(D(\mathbf{x}))] + E_{\mathbf{z}}[\log(1 - D(G(\mathbf{z})))] \tag{S1}$$

where $E_{\mathbf{x}}$ and $E_{\mathbf{z}}$ are the expected values of overall real and generated fake samples, $G(\mathbf{z})$. During training, the generator's ability to generate realistic images continually improves until the discriminator is unable to distinguish the difference between a real sample and a generated fake one. GAN-based frameworks have several desirable properties compared to other generative methods. First, GANs do not require strong assumptions regarding the form of the output probability distribution. Second, adversarial training, which uses the discriminator, allows unsupervised training of the GAN (St-Yves and Naselaris, 2018).

The variational autoencoder (VAE) proposed by Kingma and Welling (2014) is a popular generative algorithm used in neural decoding (see Figure S1 C). Similar to autoencoders, the VAE is composed of an encoder and a decoder. The encoder $q_\phi(\mathbf{z}|\mathbf{x})$, parameterized with ϕ, embeds the input $\mathbf{x}$ into a latent representation $\mathbf{z}$, and decoder $p_\theta(\mathbf{x}|\mathbf{z})$, parameterized with θ, maps the latent representation $\mathbf{z}$ back to the observation. But rather than encoding a latent vector, VAE encodes a distribution over the latent space, which makes the generative process possible. Thus, the goal of VAE is to find a distribution of the latent variable $\mathbf{z} \sim q_\phi(\mathbf{z}|\mathbf{x})$, where $q_\phi(\mathbf{z}|\mathbf{x})$ is a Gaussian distribution whose mean and standard deviation are the output of the encoder. The distribution $q_\phi(\mathbf{z}|\mathbf{x})$ is regularized to be a simple Gaussian distribution based on the Kullback-Leibler divergence between $q_\phi(\mathbf{z}|\mathbf{x})$, and the prior distribution $p(\mathbf{z})$ is assumed to follow a Gaussian distribution $\mathcal{N}(0, 1)$. Then we can sample $\mathbf{z} \sim q_\phi(\mathbf{z}|\mathbf{x})$ to generate new samples $\mathbf{x}' \sim p_\theta(\mathbf{x}|\mathbf{z})$. The learned latent representation $q_\phi(\mathbf{z}|\mathbf{x})$ approximates a true posterior distribution $p_\theta(\mathbf{z}|\mathbf{x})$, which is normally intractable. VAE loss consists of two terms:

$$\mathcal{L}(\theta, \phi; \mathbf{x}, \mathbf{z}) = \mathbb{E}_{q_\phi(\mathbf{z}|\mathbf{x})}\left[\log p_\theta(\mathbf{x} \mid \mathbf{z})\right] - D_{KL}\left(q_\phi(\mathbf{z} \mid \mathbf{x}) \| p(\mathbf{z})\right). \tag{S2}$$

The first term maximizes the reconstruction likelihood by penalizing the reconstruction error. The second term is a regularizer that enforces that the learned distribution $q_\phi(\mathbf{z}|\mathbf{x})$ is similar to the prior distribution $p(\mathbf{z})$

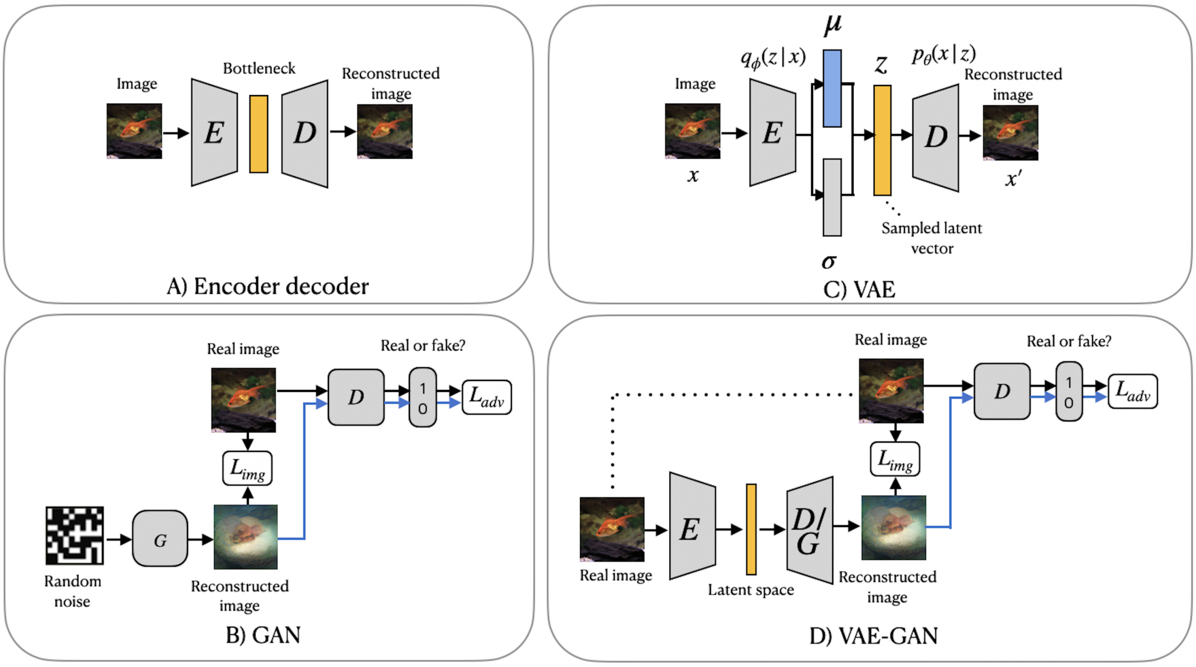

Figure S1. **(A)** Encoder–decoder architecture. **(B)** VAE. **(C)** GAN. **(D)** VAE-GAN.

by minimizing the distance error between two probability distributions measured by the Kullback–Leibler divergence.

VAE-GAN is a hybrid model by Larsen et al. (2016) integrates both the VAE and GAN. VAE-GAN combines VAE to produce compressed latent features **z** and GAN discriminator, which learns to discriminate between fake and real images. The VAE decoder and GAN generator are combined into one. Figure S1 D illustrates the VAE-GAN framework. The VAE-GAN loss is computed as follows:

$$\mathcal{L}_{VAE-GAN} = \mathcal{L}_{Dis} + D_{KL}\left(q_\phi(\mathbf{z} \mid \mathbf{x}) \| p(\mathbf{z})\right) + \mathcal{L}_{GAN}. \tag{S3}$$

Compared to the VAE loss in equation S2, VAE-GAN loss includes $\mathcal{L}_{GAN}$ loss, and the reconstruction loss $\mathcal{L}(\mathbf{x}, \mathbf{x}')$ is replaced with a reconstruction error expressed using the intermediate features of the GAN discriminator $\mathcal{L}_{Dis}$.

2 RECONSTRUCTED IMAGE RESOLUTIONS

Reconstructed image resolutions reported by surveyed works for natural image datasets are shown in Table S1.

3 VISUAL INSPECTION

For `vim-1` dataset, the `SeeligerDCGAN`, `StYvesEBGAN`, and `QiaoGAN-BVRM` methods used GAN as natural image prior to generate natural-looking images. However, low-resolution reconstructions of `StYvesEBGAN` can only capture high-level image features such as the line of the horizon but do not preserve low-level details of the stimuli. The high-resolution samples generated from `SeeligerDCGAN` and `QiaoGAN-BVRM` are more realistic and capture the high-level attributes of the image. In `QiaoGAN-BVRM`, the prior decoded categories play a central role in reconstructing high-level semantic content. For example, the reconstruction of the image in the first row of Figure S2 captures the

Table S1. Reconstructed image resolutions reported by surveyed works for natural image datasets.

Reference	Dataset	Reconstructed image size
Seeliger et al. (2018)	vim-1<br>GOD	64 x 64
St-Yves and Naselaris (2018)	vim-1	32 x 32
Shen et al. (2019b)	DIR	224 x 224
Shen et al. (2019a)	DIR	227 x 227
Beliy et al. (2019)	GOD<br>vim-1	112 x 112
VanRullen and Reddy (2019)	Faces	128 x 128
Gaziv et al. (2020)	GOD<br>vim-1	112 x 112
Qiao et al. (2020)	vim-1	128 x 128
Fang et al. (2020)	DIR	256 x 256
Mozafari et al. (2020)	GOD	256 x 256
Ren et al. (2021)	GOD	100 x 100

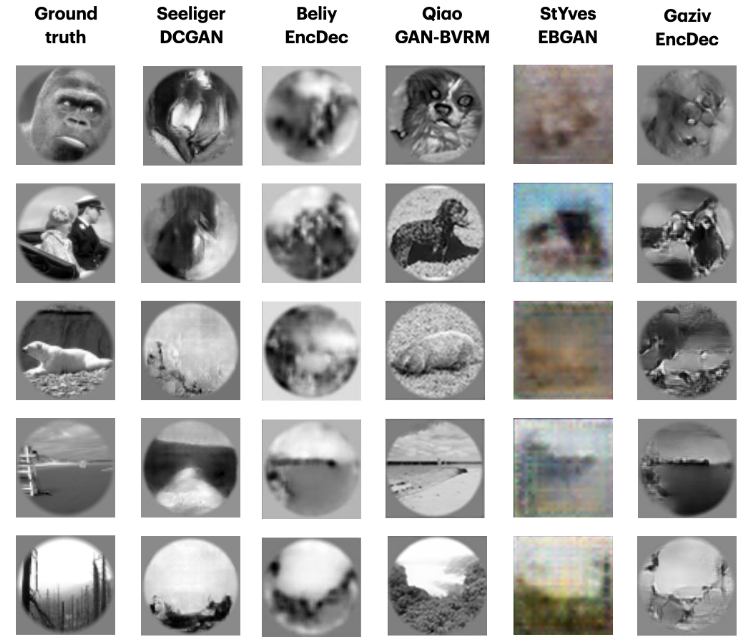

Figure S2. Visual comparison of methods on `vim-1` dataset for subject 1. The reconstructions for all methods are provided by the authors or reported in the original papers. The reconstructions for `BeliyEncDec` (Beliy et al., 2019), `QiaoGAN-BVRM` (Qiao et al., 2020), and `StYvesEBGAN` (St-Yves and Naselaris, 2018) were taken from the original papers. `SeeligerDCGAN` uses the average of the stimuli representations for the three subjects.

facial features of the object. However, in several other cases, the reconstructed object categories do not correspond to the true ones, as shown in the second row of Figure S2 (a reconstruction of "dog" object for stimuli containing a "human"). The authors attribute this to the inconsistency between image categories in `vim-1` stimuli images and ImageNet, which was used for pretraining the model. `BeliyEncDec`, `GazivEncDec`, and `StYvesEBGAN` do not clearly reconstruct identifiable objects, although their performance is the best in preserving the shape and global layout.

Table S2. One-to-one comparative evaluation across the methods on natural images from the `DIR` dataset (Shen et al., 2019b). The best results are presented in **bold**. The methods that scored the best on ≥ 2 metrics are shown in **bold**. $\downarrow$ indicates that the lower the better, and $\uparrow$ indicates the higher the better.

Subject	Method	MSE ↓	PCC ↑	SSIM ↑	PSM ↓
S1	ShenDNN (Shen et al., 2019b)	0.11±0.05	0.28±0.26	0.11±0.05	0.93±0.09
	ShenDNN+DGN (Shen et al., 2019b)	0.10±0.05	**0.32±0.29**	0.26±0.13	0.70±0.08
	ShenGAN (Shen et al., 2019a)	**0.09±0.04**	0.31±0.26	0.26±0.14	**0.66±0.06**
	BeliyEncDec (Beliy et al., 2019)	**0.09±0.04**	**0.32±0.22**	**0.31±0.13**	0.88±0.08
	FangSSGAN (Fang et al., 2020)	0.10±0.06	0.21±0.27	0.22±0.12	0.70±0.07
S2	ShenDNN (Shen et al., 2019b)	0.11±0.04	0.27±0.25	0.10±0.04	0.94±0.09
	ShenDNN+DGN (Shen et al., 2019b)	0.10±0.05	**0.29±0.29**	0.25±0.12	0.71±0.08
	ShenGAN (Shen et al., 2019a)	**0.09±0.04**	0.26±0.24	0.24±0.14	**0.68±0.06**
	BeliyEncDec (Beliy et al., 2019)	0.12±0.05	0.22±0.24	**0.30±0.14**	0.87±0.09
	FangSSGAN (Fang et al., 2020)	**0.09±0.04**	0.22±0.24	0.19±0.11	0.70±0.07
S3	ShenDNN (Shen et al., 2019b)	0.11±0.05	0.29±0.25	0.10±0.04	0.94±0.08
	ShenDNN+DGN (Shen et al., 2019b)	**0.09±0.06**	0.30±0.30	0.25±0.13	0.71±0.08
	ShenGAN (Shen et al., 2019a)	**0.09±0.05**	**0.31±0.24**	0.25±0.14	**0.66±0.06**
	BeliyEncDec (Beliy et al., 2019)	**0.09±0.04**	**0.31±0.24**	**0.31±0.14**	0.88±0.10
	FangSSGAN (Fang et al., 2020)	0.10±0.05	0.21±0.25	0.18±0.10	0.69±0.06
Average result	ShenDNN (Shen et al., 2019b)	0.11±0.05	0.28±0.25	0.11±0.05	0.94±0.01
	ShenDNN+DGN (Shen et al., 2019b)	0.10±0.05	**0.30±0.29**	0.25±0.13	0.71±0.00
	ShenGAN (Shen et al., 2019a)	**0.09±0.04**	0.29±0.25	0.25±0.14	**0.67±0.01**
	BeliyEncDec (Beliy et al., 2019)	0.10±0.04	0.28±0.23	**0.30±0.13**	0.87±0.01
	FangSSGAN (Fang et al., 2020)	0.10±0.05	0.21±0.25	0.20±0.11	0.70±0.00

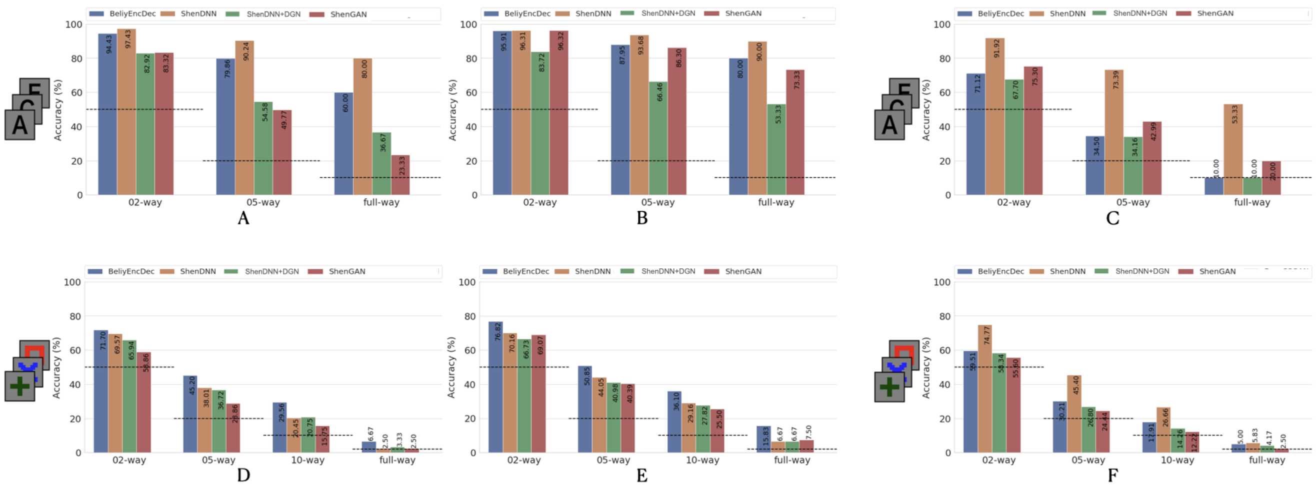

Figure S3. Average n-way accuracy results computed across subjects using MSE, PCC, and SSIM metrics on different datasets from the `DIR`: alphabetical letters (first row), and artificial shapes (second row). The horizontal dashed lines indicate the chance level for each metric. The full-way comparison corresponds to using all the images in the test set, that is, 50 natural images, 10 images in alphabetical letters, and 40 images in artificial shapes from the `DIR`).

4 COMPARATIVE ONE-TO-ONE EVALUATION ON NATURAL IMAGES

Comparative evaluation using one-to-one and pairwise settings across the methods on natural images from the `DIR` dataset (Shen et al., 2019b) is shown in Table S2.

5 GENERALIZATION TO ALPHABETICAL LETTERS AND ARTIFICIAL SHAPES

Other than natural images, `DIR` dataset contains alphabetical letters and artificial shapes images, which were originally designed to be used only for inference by a model pretrained on natural image data (Shen et al., 2019b,a). The ability of the models to generalize to artificial images from different image domains can also indicate that the reconstruction model does not simply match to exemplars (Shen et al., 2019b). In particular, similar to Shen et al. (2019a,b), we test whether the knowledge acquired from natural image reconstruction from fMRI can be transferred to the task of reconstructing shapes and letters. We perform the evaluation on shapes, and letters from the `DIR` based on the metrics: MSE, PCC, and SSIM.

In addition to running the generalizability experiment for `ShenDNN+DGN` (Shen et al., 2019b) and `ShenGAN` (Shen et al., 2019a), which demonstrated their generalization power, we ran pretrained `BeliyEncDec` (Beliy et al., 2019) on alphabetical letters and artificial shapes images from `DIR`. Generalizing the model trained on natural images to different data is challenging but necessary to ensure that the model reconstructs images from fMRI activity rather than memorizing or making matches to the stimuli (Shen et al., 2019b).

The visual reconstructions of samples from alphabetical letters and artificial shapes are presented in Figures S4 A and B. For alphabetical letters, `ShenDNN+DGN` and `ShenGAN` produce reconstructions with clear contours and backgrounds. For the artificial shapes dataset, the images were successfully reconstructed by all methods, but the reconstruction of red-colored shapes was particularly clear in `BeliyEncDec`. In reconstructions for both artificial shapes and alphabetical letters, `BeliyEncDec` (Beliy et al., 2019) produces background details not present in the original stimuli, as well as greater diversity in colors between images.

The qualitative and quantitative results presented in terms of one-to-one and pairwise MSE, PCC, and SSIM for alphabetical letters and artificial shapes are shown in Table S3 and Table S4. Three methods, `ShenDNN`, `ShenGAN`, and `BeliyEncDec`, perform the best on alphabetical letters images across at least two metrics. Similar to natural images, nonGAN methods, namely `ShenDNN` and `BeliyEncDec`, outperform other methods on low-level metrics. For artificial shapes, `BeliyEncDec` (Beliy et al., 2019) outperformed other methods on four lower-level metrics. `ShenDNN` (Shen et al., 2019b) performed significantly better on pairwise and 2-way SSIM, achieving a score of approximately 74.70%.

Overall, the performance of all n-way metrics is better for alphabetical letters compared to artificial shapes from `DIR`. The highest scores on alphabetical letters were achieved by `ShenDNN` on 2-way MSE and 2-way PCC metrics, (97.43% and 96.31%, respectively).

6 REGIONS OF INTEREST IN FMRI

A common approach to fMRI analysis involves extracting signals from sections of the brain, called regions of interest (ROIs). The hierarchical nature of information processing in the visual cortex is shown in (Poldrack and Farah, 2015). According to this finding, low-level image features (such as shapes and orientations) show a high correlation with the brain response signal in lower ROIs—the lower visual cortex (LVC). In contrast, high-level image features (such as semantic object categories) are shown to be encoded in higher ROIs, that is, the higher visual cortex (HVC). The lower visual cortex (LVC) consists of lower-order visual areas (V1, V2, and V3). Higher-order visual areas include the lateral occipital complex (LOC), the fusiform face area (FFA), and the parahippocampal place area (PPA). Table S5 shows the details of the fMRI dataset's ROIs and the number of voxels per ROI in publicly available benchmark datasets.

Table S3. Comparative evaluation across the methods on alphabetical letters from `DIR` dataset (Shen et al., 2019b). The best results are presented in **bold**. ↓ indicates that a lower value is better, and ↑ indicates a higher value is better.

		One-to-one			Pairwise		
**Subject**	**Method**	**MSE ↓**	**PCC ↑**	**SSIM ↑**	**MSE ↑**	**PCC ↑**	**SSIM ↑**
S1	ShenDNN (Shen et al., 2019b)	0.08±0.01	0.51±0.06	0.17 ± 0.01	**98.89**	**100.00**	**96.67**
	ShenDNN+DGN (Shen et al., 2019b)	0.07±0.01	**0.53±0.04**	0.5 ± 0.04	90.00	91.11	73.33
	ShenGAN (Shen et al., 2019a)	0.07±0.01	0.52±0.06	**0.54 ± 0.06**	83.33	94.44	74.44
	BeliyEncDec (Beliy et al., 2019)	**0.06±0.02**	0.41±0.08	**0.54 ± 0.06**	96.67	**100.00**	76.67
S2	ShenDNN (Shen et al., 2019b)	0.08±0.01	0.44±0.06	0.14 ± 0.02	**95.56**	88.89	**87.78**
	ShenDNN+DGN (Shen et al., 2019b)	0.07±0.01	**0.46±0.06**	0.46 ± 0.03	73.33	67.78	64.44
	ShenGAN (Shen et al., 2019a)	0.07±0.01	0.37±0.14	0.51 ± 0.08	83.33	**96.67**	68.89
	BeliyEncDec (Beliy et al., 2019)	**0.05±0.01**	0.35±0.08	**0.53 ± 0.05**	90.00	90.00	60.00
S3	ShenDNN (Shen et al., 2019b)	0.09±0.01	0.51±0.04	0.14 ± 0.01	**97.78**	**100.00**	**91.11**
	ShenDNN+DGN (Shen et al., 2019b)	0.06±0.01	**0.52±0.05**	0.46 ± 0.04	85.56	92.22	65.56
	ShenGAN (Shen et al., 2019a)	0.08±0.02	0.49±0.13	0.53 ± 0.06	83.33	97.78	82.22
	BeliyEncDec (Beliy et al., 2019)	**0.05±0.02**	0.35±0.12	**0.55 ± 0.04**	96.67	97.78	76.67
Average result	ShenDNN (Shen et al., 2019b)	0.08±0.00	0.49±0.04	0.15 ± 0.02	**97.41±1.70**	**96.30±6.41**	**91.85±4.49**
	ShenDNN+DGN (Shen et al., 2019b)	0.07±0.00	**0.51±0.04**	0.47 ± 0.02	82.96±8.63	83.70±13.80	67.78±4.84
	ShenGAN (Shen et al., 2019a)	0.07±0.01	0.46±0.08	0.53 ± 0.02	83.33±0.00	**96.30±1.70**	75.19±6.70
	BeliyEncDec (Beliy et al., 2019)	**0.06±0.01**	0.37±0.03	**0.54 ± 0.01**	94.44±3.85	95.93±5.25	71.11±9.62

Table S4. Comparative evaluation across the methods on artificial shapes from `DIR` dataset (Shen et al., 2019b). The best results are presented in **bold**. ↓ indicates that the lower the better, and ↑ indicates the higher the better.

		One-to-one			Pairwise		
**Subject**	**Method**	**MSE ↓**	**PCC ↑**	**SSIM ↑**	**MSE ↑**	**PCC ↑**	**SSIM ↑**
S1	ShenDNN (Shen et al., 2019b)	0.09±0.02	0.23±0.23	0.14±0.02	69.10	69.55	**74.49**
	ShenDNN+DGN (Shen et al., 2019b)	**0.06±0.02**	**0.28±0.28**	0.46±0.06	67.56	66.79	60.58
	ShenGAN (Shen et al., 2019a)	**0.06±0.02**	0.14±0.20	0.48±0.13	57.63	66.60	56.03
	BeliyEncDec (Beliy et al., 2019)	**0.06±0.02**	0.16±0.21	**0.60±0.08**	**72.82**	**76.35**	60.83
S2	ShenDNN (Shen et al., 2019b)	0.10±0.02	0.24±0.21	0.14±0.04	72.31	72.05	**76.54**
	ShenDNN+DGN (Shen et al., 2019b)	0.07±0.01	**0.25±0.26**	0.45±0.09	64.62	69.10	56.67
	ShenGAN (Shen et al., 2019a)	**0.06±0.02**	0.15±0.20	0.46±0.12	58.40	68.08	53.46
	BeliyEncDec (Beliy et al., 2019)	**0.06±0.02**	0.20±0.17	**0.60±0.10**	**73.21**	**76.15**	58.01
S3	ShenDNN (Shen et al., 2019b)	0.10±0.02	0.23±0.22	0.13±0.02	67.18	68.85	**73.08**
	ShenDNN+DGN (Shen et al., 2019b)	0.07±0.02	**0.25±0.28**	0.45±0.05	65.71	64.23	57.82
	ShenGAN (Shen et al., 2019a)	**0.06±0.02**	0.19±0.21	0.50±0.13	60.77	72.44	57.31
	BeliyEncDec (Beliy et al., 2019)	**0.06±0.02**	0.21±0.19	**0.60±0.07**	**69.04**	**78.01**	59.68
Average result	ShenDNN (Shen et al., 2019b)	0.10±0.00	0.23±0.00	0.14±0.00	69.53±2.59	70.15±1.68	**74.70±1.74**
	ShenDNN+DGN (Shen et al., 2019b)	0.07±0.00	**0.26±0.01**	0.45±0.01	65.96±1.49	66.71±2.44	58.35±2.01
	ShenGAN (Shen et al., 2019a)	**0.06±0.00**	0.16±0.03	0.48±0.02	58.93±1.64	69.04±3.03	55.60±1.96
	BeliyEncDec (Beliy et al., 2019)	**0.06±0.00**	0.19±0.02	**0.60±0.00**	**71.69±2.30**	**76.84±1.02**	59.51±1.42

Table S5. Details of the fMRI dataset's ROIs and the number of voxels per ROI in publicly available benchmark datasets. The subject number is specified in brackets. n/a represents information not explicitly provided.

**Reference**	**Dataset**		**ROIs**	**Voxels**
Kay et al. (2008)	`vim-1`		V1, V2, V3, V3A, V3B, V4, LO	25915 (S1), 26329 (S2)
VanRullen and Reddy (2019)	`Faces`		n/a	n/a
Horikawa and Kamitani (2017)	`Generic Object decoding`		V1, V2, V3, V4, LOC, FFA, PPA	4466 (S1), 4404(S2), 4643 (S3), 4133 (S4), 4370 (S5)
Shen et al. (2019b)	`Deep Image Reconstruction`	natural images	V1, V2, V3, V4, LOC, FFA, PPA	11726 (S1), 11114 (S2), 9919 (S3)
		artificial shapes	V1, V2, V3, V4, LOC, FFA, PPA	11726 (S1), 11114 (S2), 9919 (S3)
		alphabetical letters	V1, V2, V3, V4, LOC, FFA, PPA	11726 (S1), 11114 (S2), 9919 (S3)

REFERENCES

Beliy, R., Gaziv, G., Hoogi, A., Strappini, F., Golan, T., and Irani, M. (2019). From voxels to pixels and back: Self-supervision in natural-image reconstruction from fMRI. In *Advances in Neural Information Processing Systems 32*, eds. H. Wallach, H. Larochelle, A. Beygelzimer, F. d. Alché-Buc, E. Fox, and R. Garnett (Curran Associates, Inc.). 6517–6527

Cho, K., van Merriënboer, B., Gulcehre, C., Bahdanau, D., Bougares, F., Schwenk, H., et al. (2014). Learning Phrase Representations using RNN Encoder–Decoder for Statistical Machine Translation. In *Proceedings of the 2014 Conference on Empirical Methods in Natural Language Processing (EMNLP)* (Doha, Qatar: Association for Computational Linguistics), 1724–1734. doi:10.3115/v1/D14-1179

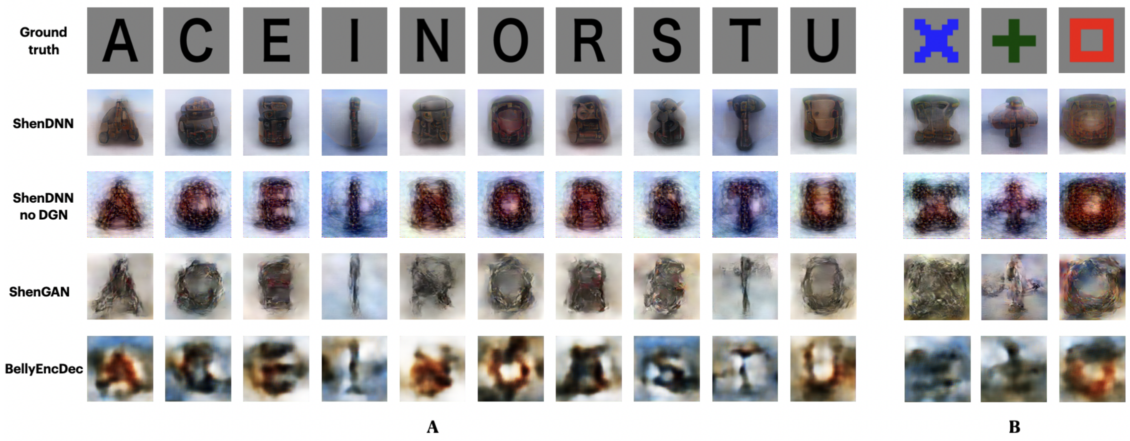

Figure S4. Reconstructed samples from generalization task: **(A)** alphabetical letters and **(B)** artificial shapes

Fang, T., Qi, Y., and Pan, G. (2020). Reconstructing Perceptive Images from Brain Activity by Shape-Semantic GAN. *Advances in Neural Information Processing Systems* 33

Gaziv, G., Beliy, R., Granot, N., Hoogi, A., Strappini, F., Golan, T., et al. (2020). *Self-Supervised Natural Image Reconstruction and Rich Semantic Classification from Brain Activity*. preprint, Neuroscience. doi:10.1101/2020.09.06.284794

Goodfellow, I. J., Pouget-Abadie, J., Mirza, M., Xu, B., Warde-Farley, D., Ozair, S., et al. (2014). Generative adversarial nets. In *Proceedings of the 27th International Conference on Neural Information Processing Systems - Volume 2* (Cambridge, MA, USA: MIT Press), NIPS'14, 2672–2680

Horikawa, T. and Kamitani, Y. (2017). Generic decoding of seen and imagined objects using hierarchical visual features. *Nature Communications* 8, 15037. doi:10.1038/ncomms15037. Number: 1 Publisher: Nature Publishing Group

Isola, P., Zhu, J.-Y., Zhou, T., and Efros, A. A. (2017). Image-To-Image Translation With Conditional Adversarial Networks. 1125–1134

Kay, K. N., Naselaris, T., Prenger, R. J., and Gallant, J. L. (2008). Identifying natural images from human brain activity. *Nature* 452, 352–355. doi:10.1038/nature06713. Number: 7185

Kingma, D. P. and Welling, M. (2014). Auto-Encoding Variational Bayes. *arXiv:1312.6114 [cs, stat]* ArXiv: 1312.6114

Larsen, A. B. L., Sønderby, S. K., Larochelle, H., and Winther, O. (2016). Autoencoding beyond pixels using a learned similarity metric. In *International Conference on Machine Learning* (PMLR), 1558–1566. ISSN: 1938-7228

Minaee, S., Boykov, Y. Y., Porikli, F., Plaza, A. J., Kehtarnavaz, N., and Terzopoulos, D. (2021). Image Segmentation Using Deep Learning: A Survey. *IEEE Transactions on Pattern Analysis and Machine Intelligence* , 1–1doi:10.1109/TPAMI.2021.3059968. Conference Name: IEEE Transactions on Pattern Analysis and Machine Intelligence

Mozafari, M., Reddy, L., and VanRullen, R. (2020). Reconstructing Natural Scenes from fMRI Patterns using BigBiGAN. In *2020 International Joint Conference on Neural Networks (IJCNN)* (Glasgow, United Kingdom: IEEE), 1–8. doi:10.1109/IJCNN48605.2020.9206960

Poldrack, R. A. and Farah, M. J. (2015). Progress and challenges in probing the human brain. *Nature* 526, 371–379. doi:10.1038/nature15692. Number: 7573 Publisher: Nature Publishing Group

Qiao, K., Chen, J., Wang, L., Zhang, C., Tong, L., and Yan, B. (2020). BigGAN-based Bayesian Reconstruction of Natural Images from Human Brain Activity. *Neuroscience* 444, 92–105. doi:10.1016/j.neuroscience.2020.07.040

Ren, Z., Li, J., Xue, X., Li, X., Yang, F., Jiao, Z., et al. (2021). Reconstructing seen image from brain activity by visually-guided cognitive representation and adversarial learning. *NeuroImage* doi:10.1016/j.neuroimage.2020.117602

Seeliger, K., Güçlü, U., Ambrogioni, L., Güçlütürk, Y., and van Gerven, M. A. J. (2018). Generative adversarial networks for reconstructing natural images from brain activity. *NeuroImage* 181, 775–785. doi:10.1016/j.neuroimage.2018.07.043

Shen, G., Dwivedi, K., Majima, K., Horikawa, T., and Kamitani, Y. (2019a). End-to-End Deep Image Reconstruction From Human Brain Activity. *Frontiers in Computational Neuroscience* 13. doi:10.3389/fncom.2019.00021. Publisher: Frontiers

Shen, G., Horikawa, T., Majima, K., and Kamitani, Y. (2019b). Deep image reconstruction from human brain activity. *PLOS Computational Biology* 15, e1006633. doi:10.1371/journal.pcbi.1006633. Number: 1 Publisher: Public Library of Science

St-Yves, G. and Naselaris, T. (2018). Generative Adversarial Networks Conditioned on Brain Activity Reconstruct Seen Images. In *2018 IEEE International Conference on Systems, Man, and Cybernetics (SMC)*. 1054–1061. doi:10.1109/SMC.2018.00187. ISSN: 2577-1655

VanRullen, R. and Reddy, L. (2019). Reconstructing faces from fMRI patterns using deep generative neural networks. *Communications Biology* 2, 1–10. doi:10.1038/s42003-019-0438-y. Number: 1 Publisher: Nature Publishing Group
